# The *nanos1* gene was duplicated in early Vertebrates and the two paralogs show different gonadal expression profiles in a shark

**DOI:** 10.1038/s41598-018-24643-1

**Published:** 2018-05-02

**Authors:** Laura Gribouval, Pascal Sourdaine, Jean-Jacques Lareyre, Johanna Bellaiche, Florence Le Gac, Sylvie Mazan, Cécile Guiardiere, Pierrïck Auvray, Aude Gautier

**Affiliations:** 1 Normandie University, UNICAEN, Sorbonne Universités, MNHN, UPMC University Paris 06, UA, CNRS, IRD, Biologie des Organismes et Ecosystèmes Aquatiques (BOREA), CS14032, 14032 CAEN Cedex 5, France; 2 KELIA, Parc Technopolitain Atalante Saint Malo, 35400 Saint Malo, France; 3 INRA UPR1037, Laboratory of Fish Physiology and Genomics, BIOSIT, Ouest-Genopole, Campus de Beaulieu 35042 Rennes, France; 4 CNRS-UPMC-Sorbonne Universités, UMR 7232, Observatoire océanologique, 66650 Banyuls sur mer, France

**Keywords:** Phylogenetics, Reproductive biology

## Abstract

Nanos are RNA-binding proteins playing crucial roles in germ cell development and maintenance. Based on phylogenetic and synteny analyses, this study reveals that *nanos1* gene has undergone multiple duplications and gene copies losses in Vertebrates. Chondrichthyan species display two *nanos1* genes (named *nanos1A*/*1B*), which were both retrieved in some Osteichthyes at basal positions in Sarcopterygii and Actinopterygii lineages. In contrast, Teleosts have lost *nanos1A* but duplicated *nanos1B* leading to the emergence of two ohnologs (*nanos1Ba/1Bb*), whereas Tetrapods have lost *nanos1B* gene. The two successive nanos gene duplications may result from the second and third whole genome duplication events at the basis of Vertebrates and Teleosts respectively. The expression profiles of *nanos1A* and *nanos1B* paralogs were characterized in the dogfish, *Scyliorhinus canicula*. *Nanos1A* was strongly expressed in brain and also localized in all germ cell types in the polarized testis. In contrast, *nanos1B* was detected in testis with the highest expression in the germinative zone. In addition, Nanos1B protein was predominantly located in the nuclei of male germinal cells. In the ovary, both paralogs were detected in germinal and somatic cells. Our study opens new perspectives concerning the complex evolution of *nanos1* paralogs and their potential distinct roles in Vertebrates gonads.

## Introduction

The Nanos proteins family regroups highly conserved RNA-binding proteins in higher eukaryotes implicated in germ cell development and maintenance. Nanos family is characterized by two specific Cys-Cys-His-Cys zinc finger motifs at the carboxy-terminal region which present a regular spacing between the Cys and the His residues. These motifs are indispensable for Nanos function^1^. They can bind to the 3′ untranslated region (3′UTR) of target messenger RNAs (mRNAs) in order to regulate gene expression post-transcriptionally with no sequence specificity but by potential electrostatic interactions with the phosphate backbone of RNA^2,3^. For example, in the mouse, NANOS2 plays a role in meiosis suppression by preventing Stra8 expression in male foetal gonads^4^.

Nanos has been first identified in *Drosophila melanogaster* as a maternal gene crucial for abdomen formation^5^ and for germ cells implantation^6^. In Vertebrates, three *Nanos* paralogous genes were described. In *Xenopus*, knockdown of *Nanos1* induced the loss of Primordial Germ Cells (PGCs)^7^ and in zebrafish the absence of Nanos3 disturbed PGCs migration and decreased their number^8^. In mice, the suppression of Nanos3 expression in PGC resulted in the complete loss of germ cells in both sexes^9^. NANOS2 has also been identified as a key stem cell regulator in Spermatogonial Stem Cells (SSC) of mature individuals by maintaining the stem cell fate during spermatogenesis in mice^10^. A role of this gene in Germinal Stem Cells maintenance may be evolutionarily conserved as Nanos2 is specifically expressed both in oogonia and in spermatogonia of adult medaka^11^ and in a subpopulation of undifferentiated A spermatogonia in juvenile and spermiating male trout^12^. Taken together, these data identify *Nanos* as primordial genes with highly conserved functions for both, the migration of the germinal cells and their maintenance in adults.

In Vertebrates, *Nanos* paralogous genes were associated to partial redundancies and specific functional evolutions. For example, in mice, *Nanos1* was predominantly expressed in the central nervous system and in adult gonads but *Nanos1*^−/−^ mice developed normally and were fertile^13^. In contrast, a mutation of *NANOS1* led to male infertility in human^14^. On the other hand, while NANOS2 and NANOS3 were crucial for the germ cell lineage establishment in mice^9^, mutations of these genes in humans did not cause infertility^15,16^.

In teleostean fish, two *nanos1* genes were generated, probably during the Teleost specific whole genome duplication (3R). In medaka, *in situ* hybridization revealed that the two forms of *nanos1* gene showed specific expression patterns in developing brain and sensory organs. These two transcripts showed a differential expression in the gonad: *nanos1a* was expressed in the somatic cells surrounding oocytes and in male meiotic cells unlike n*anos1b*, which was not detectable^11^. All these studies illustrate the complexity and the diversity of *nanos* gene expression patterns and functions through Vertebrates evolution.

Because of their phylogenetic position as sister group of Osteichthyes, Chondrichthyes represent models of interest to gain insight into gene evolution in jawed Vertebrates which appeared after the two rounds of whole genome duplication in Vertebrates (1R and 2R). For this purpose, genomic and transcriptomic data are progressively available in holocephalans, in the elephant shark, *Callorhinchus milii*^17^ and in Elasmobranchs, in the whale shark *Rhincodon typus*^18^ and in the little skate *Leucoraja erinacea*^19,20^. Unpublished transcriptome libraries were also produced from another elasmobranch, the small-spotted dogfish, *Scyliorhinus canicula*^21,22^. Molecular and cellular data concerning the reproduction of these species are very limited whereas one quarter of Chondrichthyes are threatened according to the International Union for the Conservation of Nature (IUCN) Red List criteria^23^. Our model, *Scyliorhinus canicula*, is a non-vulnerable shark present in abundance in the English Channel. Females present a unique ovary containing follicles at different stages of maturation. Males display polarized testes, organized in cysts, allowing a precise description of the expression pattern of specific genes during the spermatogenic progression. Indeed, the testis can be subdivided in five testicular zones specific to the different areas of spermatogenesis: the zone A0 containing the potential Spermatogonial Stem Cells (SSC) in a dense somatic tissue, and the other areas made of cysts containing respectively differentiating spermatogonia (zone A-), spermatocytes (zone B), round spermatids (zone C) and elongated spermatids (zone D)^24^. This particular organization allowed us to evaluate more precisely the stage-specific expression of particular transcripts and proteins during spermatogenesis^25,26^.

In this study, we identified in *Scyliorhinus canicula* transcriptomes two sequences which both segregated with the Nanos1 proteins subfamily. This finding led us to investigate their origin and evolution in Vertebrates using phylogenetic reconstructions and analyses of syntenic chromosomal fragments. These analyses revealed multiple duplications and losses of *nanos1* gene copies during Vertebrates evolution. The expression profile of these two paralogs was next characterized in a representative of basal Gnathostomes, the dogfish, in a panel of tissue by RT-PCR and in the gonads by immunohistochemistry and *in situ* hybridization on mature males and females. The two gene copies, termed *nanos1A* and *nanos1B*, were both detected in germinal and somatic cells in the ovary, whereas they showed different germinal distribution in testis. This work shows that *nanos1* genes in Sarcopterygians and Teleosts provide an example of hidden paralogy, and describes for the first time the distinct expression profiles of the two *nanos1A* and *nanos1B* paralogs using a shark.

## Results

### Multiple *nanos1* paralogs exist in Vertebrates

**Figure 1:**
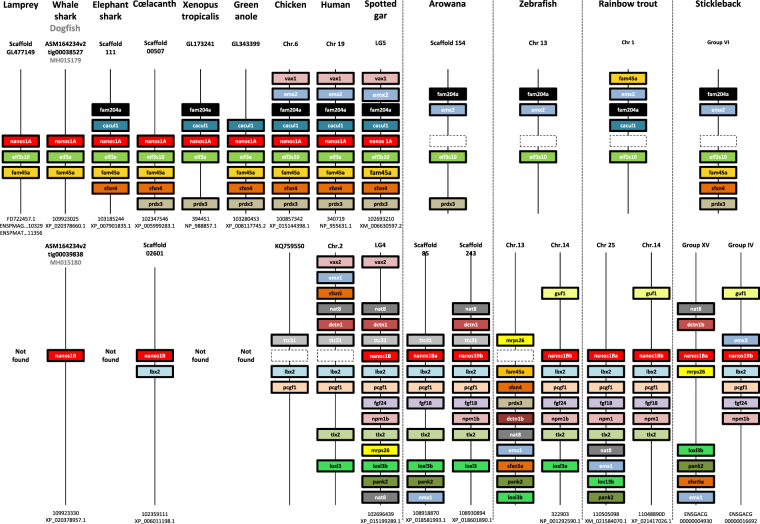
Gene synteny comparisons provide evidences that multiple *nanos1* gene duplications and losses occurred in vertebrates Three *nanos1* paralogs and their neighbouring genes showed syntenic genomic locations through Vertebrates. In the top panel, genes in the vicinity of a first copy, named *nanos1A*, were mapped. The figure was not drawn to scale. Each gene was represented by a specific coloured box. The name of each scaffold or chromosome harbouring the synteny is indicated at the top for each species whereas *nanos1* gene copy and protein accession numbers are detailed at the bottom. Although the structure of the chromosomal fragment was well conserved during the evolution, the *nanos1A* gene copy is not observed in the genome of Teleost fish as symbolized by the dotted boxes. In the bottom panel, the genomic environment of the second *nanos1* gene copy, termed *nanos1B*, was similarly represented. This different syntenic chromosomal fragment was not found in lamprey suggesting its apparition in Gnathostomata. Both *nanos1* paralogs (*nanos1A* and *nanos1B*) were detected In Chondrichthyes (dogfish and whale shark) and in Osteichthyes, respectively at the basis of Sarcopterygii (coelacanth) and of Actinopterygii (spotted gar). In contrast, *nanos1B* gene was not found in elephant shark, xenopus, green anole, chicken and human, suggesting its loss in these species. Teleosts showed two *nanos1B* gene copies carried by similar but distinct chromosomal fragments. The two *nanos1* paralogs in Teleosts were re-named *nanos1Ba* and *nanos1Bb*. Note that zebrafish is an atypical fish species because its genome does not harbour *the nanos1Ba* gene copy as indicated by a spotted box.

**Figure 2:**
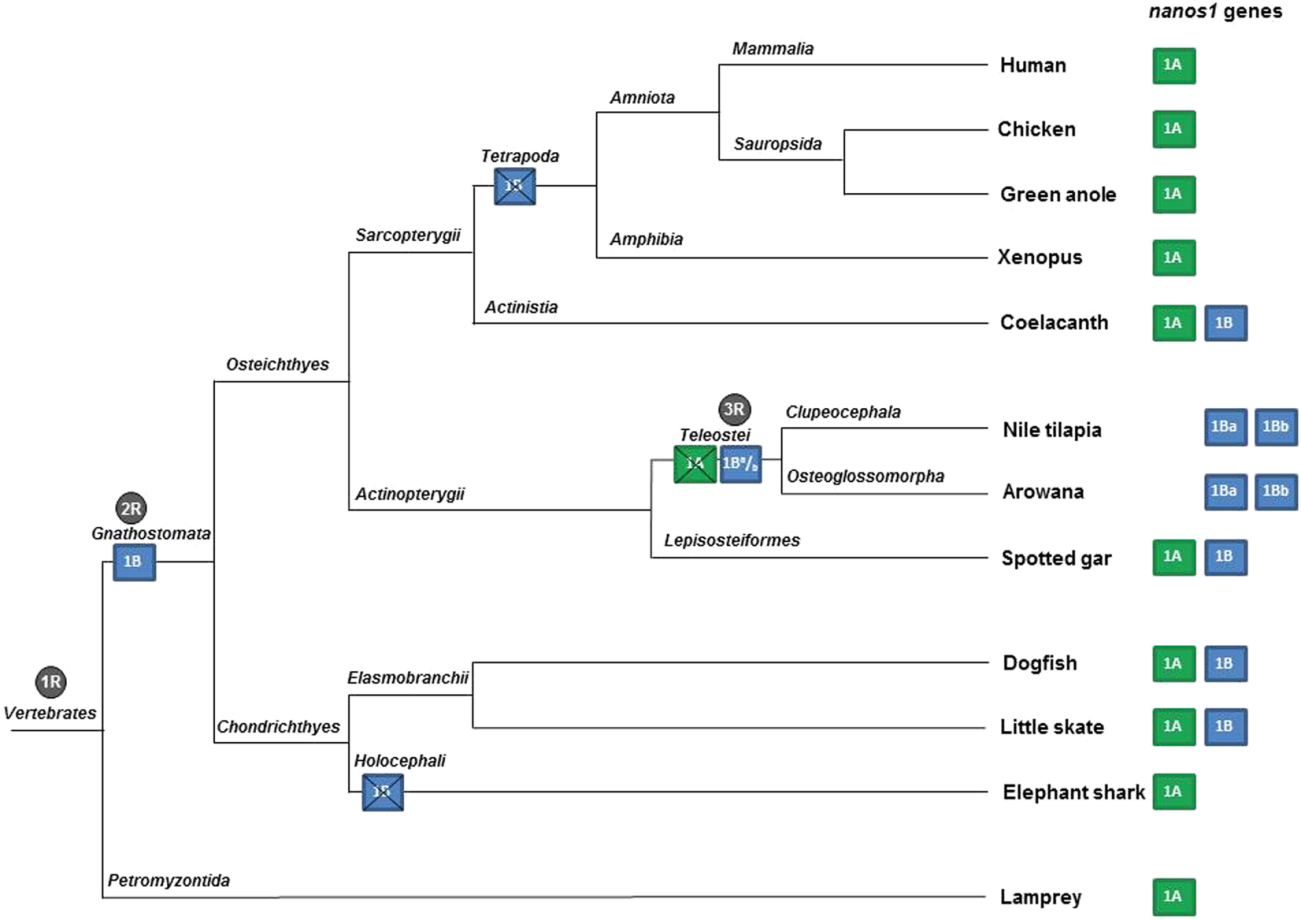
Hypothetical model of *nanos1* gene duplications and copy losses during Vertebrate evolution. Based on *nanos1* syntenies and phylogenetic trees, two rounds of duplication of the ancestral lamprey *nanos1* gene were observed. At the basis of Vertebrates, the sea lamprey displays only the ancestral *nanos1A* gene copy. The first gene duplication giving rise to the *nanos1B* paralog may have occurred following the second genome duplication (2R) at the basis of Gnathostomata (box 1B). Chondrichthyan species (shark and ray) have kept both gene copies except the Holocephali (chimaera). In Osteichthyes, both paralogs were found in basal Sarcopterygii (coelacanth) but *nanos1B* was lost by Tetrapoda (crossed box 1B). Both *nanos1A* and *nanos1B* paralogs were found in basal Actinopterygii (spotted gar) but *nanos1A* was lost in Teleostei (crossed box 1A). Coinciding with the third round of whole genome duplication (3R), *nanos1B* gene copy would have undergone a second duplication in a Telelost ancestor giving rise to *nanos1Ba* and *nanos1Bb gene copies* (box 1B^a^/_b_). The *nanos1* paralogs present in the different species are summarized on the right.

The identification of *nanos* sequences in *Scyliorhinus canicula* was carried out from different dogfish cDNA banks generated from ovary, testis and a pool of embryos, juveniles and adult tissues. Two different complete cDNA sequences were found using the TBLASTN algorithm and mouse NANOS proteins as queries. A phylogenetic analysis showed that both dogfish sequences segregated with Nanos1. A first sequence showed highest similarity with the mammalian NANOS1 proteins (termed Nanos1A) whereas the second one was similar to the teleostean Nanos1 proteins (termed Nanos1B). This result raised the question of the timing of this gene duplication relative to the Gnathostomes radiation. In order to address this issue, *nanos1* sequences were first searched in other chondrichthyan species. Concerning Elasmobranchs, both *nanos1* gene copies were found in the whale shark *Rhincodon typus* genome and in the little skate *Leucoraja erinacea* genome and transcriptome (SkateBase: *nanos1A*, LS-transcript-ctg90798; *nanos1B partial sequences*, LS-transcript-ctg92973 and LS-transcriptB2-ctg57931). In the chimaera *Callorhinchus milii*, only *nanos1A* was found. The search for *Nanos1* genes was then extended to other vertebrates and additional *nanos1* gene copies were determined based on phylogenetic and genomic environment analyses. The conserved neighbouring genes of *nanos1A* and *nanos1B* were mapped in the genome of representative vertebrate species: Agnatha (sea lamprey), Chondrichthyes (dogfish, whale shark, chimaera/elephant shark) and Osteichthyes, including Sarcopterygii (coelacanth, *Xenopus tropicalis*, green anole, chicken and human) and Actinopterygii (spotted gar, arowana, zebrafish, rainbow trout and stickleback) (Fig. 1). The *nanos1A* synteny was found in Agnatha (sea lamprey), Chondrichthyes and Osteichthyes but Teleosts have lost *nanos1A* gene (dotted box). The *nanos1B* synteny was not found in sea lamprey, chimaera (elephant shark), *Xenopus tropicalis* and green anole. The synteny was present but lacked *nanos1B* gene in chicken and human (dotted box). The genomic environments of the two copies of *nanos1* previously described in Teleost fish both corresponded to *nanos1B* synteny. Note that zebrafish *nanos1Ba* synteny has been submitted to chromosomal rearrangements leading to the loss of this gene but the introduction of three successive genes from *nanos1A* synteny. Interestingly, *nanos1A* and *nanos1B* paralogs were both found in chondrichthyan species (ray and sharks) as well as in osteichthyan species at the basis of Sarcopterygii (coelacanth) and Actinopterygii (spotted gar). In addition, *nanos1* syntenies showed common paralogs of *sfxn* and *emx* (*sfxn4* and *emx2* around *nanos1A; sfxn5* and *emx1* in the vicinity of *nanos1B*), suggesting that both *nanos1A* and *nanos1B* gene copies originate from the duplication of an ancestral chromosomal fragment. These results were summarized in a hypothetical model of *nanos1* evolution in Fig. 2. Concerning Nanos2 and Nanos3, their genomic environments differ from *nanos1* genes syntenies and from each other. Few genes neighbouring *nanos2* are conserved between basal Actinopterygii (spotted gar) and Sarcopterygii (coelacanth and human) but the syntenic relationship becomes elusive in Teleosts (Supplementary Figure S1). In contrast, the genomic environment of *nanos3* is remarkably conserved from sharks to human and Teleosts (Supplementary Figure S2).

### The *nanos1* paralogous genes displayed different evolutionary rates

A phylogenetic tree was constructed using Nanos protein sequences of representative Vertebrate species including four dogfish *nanos* sequences identified in the present study (Supplementary Figure S3). Nanos1A and Nanos1B paralogous proteins segregated separately as expected. Due to their high inter-species conservation, Nanos1B sequences of Osteichthyes were compressed in this global tree. Another secondary phylogenetic tree was built to further investigate the *nanos1B* paralogs in Teleosts. Nanos1Ba and Nanos1Bb sequences segregated together inside different clades corresponding to Acanthopterygians, Cypriniformes, Salmoniformes and Osteoglossiformes respectively (shaded boxes), supporting the hypothesis that *nanos1Ba* and *nanos1Bb* are ohnologs resulting from the duplication of an ancestral actinopterygian *nanos1B* gene. The Nanos1 paralogous proteins all harbour the two Nanos family CCHC zinc finger motifs at their carboxy terminal conserved end (asterisks, Supplementary Figure S4). For amino acid alignment of Nanos1 sequences, representative Vertebrate species were selected among Chondrichthyes (dogfish, chimaera) and Osteichthyes, including Sarcopterygii (coelacanth, chicken and human) and Actinopterygii (spotted gar, arowana and salmon). Nanos1B proteins show a higher conservation compared to Nanos1A proteins. For example, coelacanth Nanos1A presents only a mean of 7% identity and 16% similarity with the other selected Nanos1A whereas Nanos1B presents respectively 34% identity and 43% similarity with the other selected Nanos1B sequences. In summary, *nanos1A* and *nanos1B* paralogs show different evolutionary rates after duplication of the ancestral gene but a different gene copy was retained finally in Teleosts (3R-ohnologs *nanos1Ba* and *nanos1Bb*) and in Tetrapods (*nanos1A*).

### *nanos1A* and *nanos1B* have different tissue expression patterns in the dogfish

**Figure 3:**
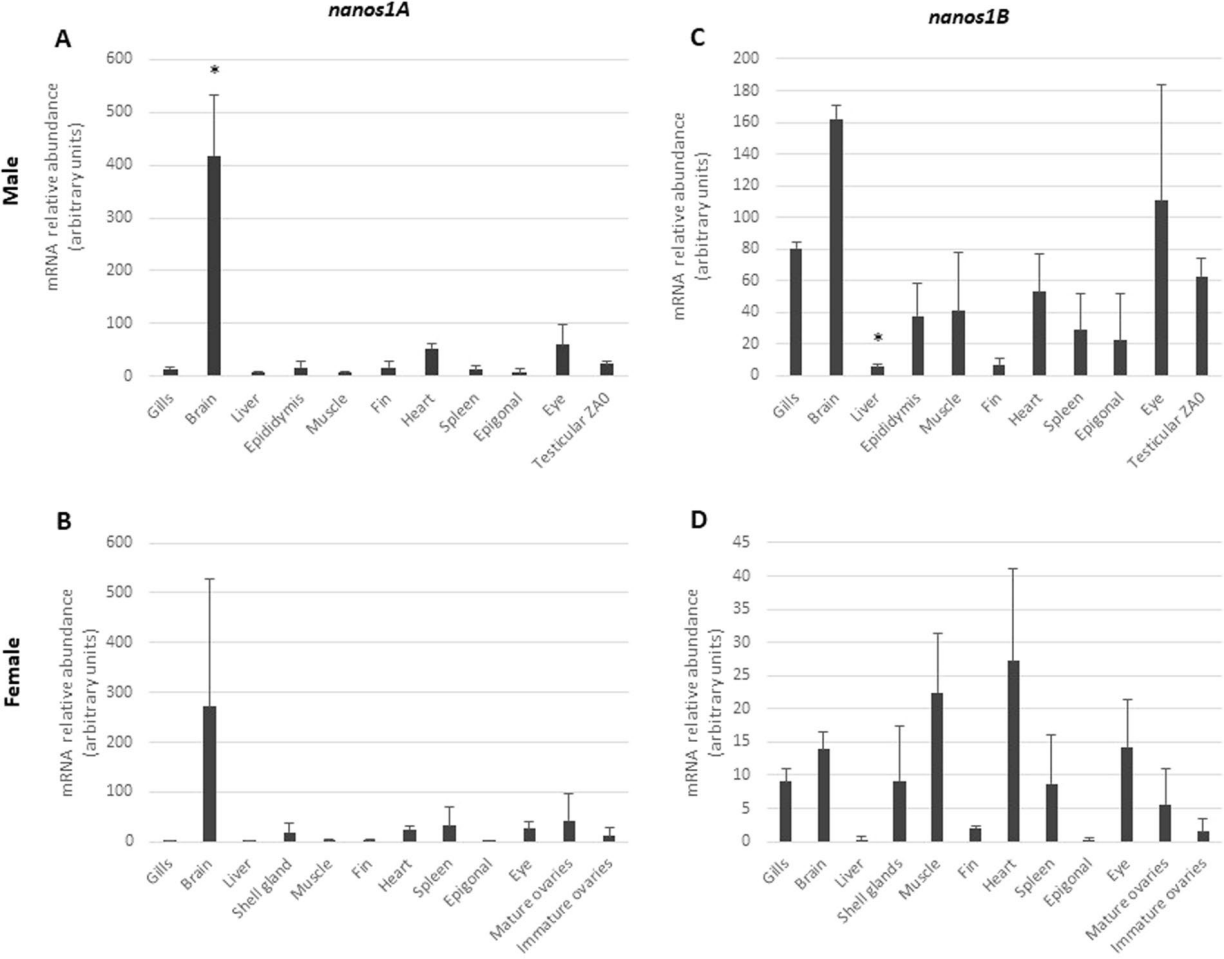
nanos1A and na*nos1B* transcripts show distinct tissue distributions in male and female *S. canicula*. Messenger RNA relative abundances of *nanos1A* (**A**,**B**) and *nanos1B* (**C**,**D**) were quantified in panels of tissues in male (**A**,**C**) and female **(B**,**D**) dogfish by real-time PCR. Data were normalized using *5S rRNA*. In both sexes, *nanos1A* showed a preferential expression in brain (**A**,**B**) whereas *nanos1B* showed a relatively ubiquitous expression pattern (**C**,**D**). Mature ovaries sampled from adult females and containing vitellogenic oocytes showed no significant difference of expression levels compared to immature ovaries sampled from pre-adult females and containing previtellogenic oocytes only (**B**,**D**). Data are presented as mean + SD (N = 3). Differences in gene expression were evaluated using one-way analysis of variance followed up with Games-Howell test. Significant differences (p ≤ 0.05) in gene expression compared to the testicular zone A0 or the immature ovary are indicated by asterisks (*).

**Figure 4:**
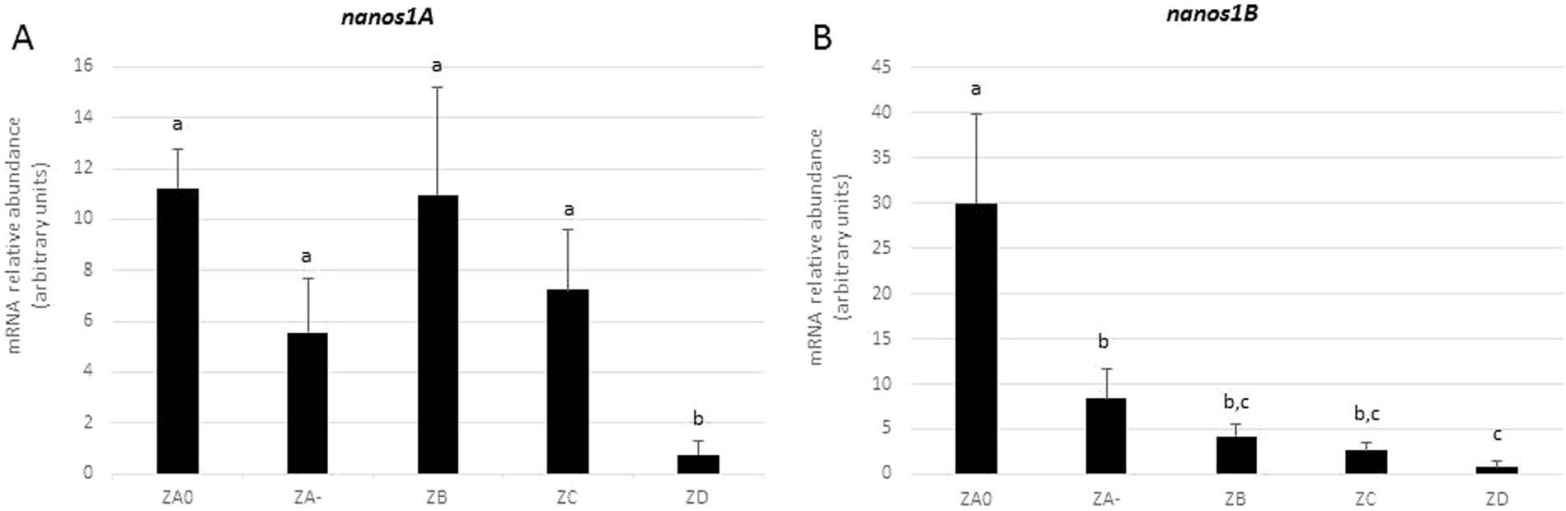
Specific expression patterns of *nanos1A* and *nanos1B* in dogfish testicular zones. The relative abundance of *nanos1A* and *nanos1B* mRNAs in the five testicular zones was measured by RT-PCR and normalized with *5S rRNA*. Dogfish polarized testis was dissected in five zones from its dorsal to its ventral side: ZA0, germinative zone containing SSCs; ZA-, cysts with spermatogonia; ZB, meiotic zone; ZC, cysts with round-spermatids; and ZD, cysts with elongated spermatids and zone of cyst resorption. The *nanos1A* transcript was detected in all zones except in zone D where it was almost undetectable. In contrast, *nanos1B* showed a marked progressive decrease of its expression after the germinative zone, ZA0. Data are presented as mean + SD (N = 4). Differences in gene expression were evaluated using one-way analysis of variance followed up with Games-Howell test. Testicular zones sharing no letter in common show significantly different expression levels.

The tissue distribution of the two paralogous *nanos1A* and *nanos1B* genes was assessed in male and female *S. canicula* by real-time PCR on a panel of tissues including gills, brain, liver, muscle, fin, heart, spleen, epigonal tissue, eye and sex-specific organs such as shell gland, ovary, epididymis and testis (Fig. 3). In all males and a female, *nanos1A* mRNA was detected at a very high level in brain in comparison with other tissues (Fig. 3A and B). In contrast, *nanos1B* showed a relatively ubiquitous expression pattern with an expression that remained significantly higher in the brain of males, but not of females, comparatively to the other tissues, except eyes (Fig. 3C and D). No significant difference of expression of *nanos1A* and *nanos1B* was detected between immature and mature ovaries (Fig. 3B and D). In testicular zones, the expression of *nanos1A* was detected at a relative constant level during spermatogenesis, between the germinative zone (ZA0) and the zones containing cysts with spermatogonia (ZA-), spermatocytes (ZB) and early spermatids (ZC), and was almost undetectable in zone D containing cysts with late spermatids (Fig. 4A). Concerning *nanos1B*, a clear progressive decrease of its expression was observed from the germinative zone (ZA0) to zone D (Fig. 4B). Our data suggested that nanos1A and nanos1B might have a distinct expression profile in the testis.

### In testis, *nanos1B* mRNA was restricted to spermatogonia contrary to *nanos1A* expressed in germ cells at all stages

**Figure 5:**
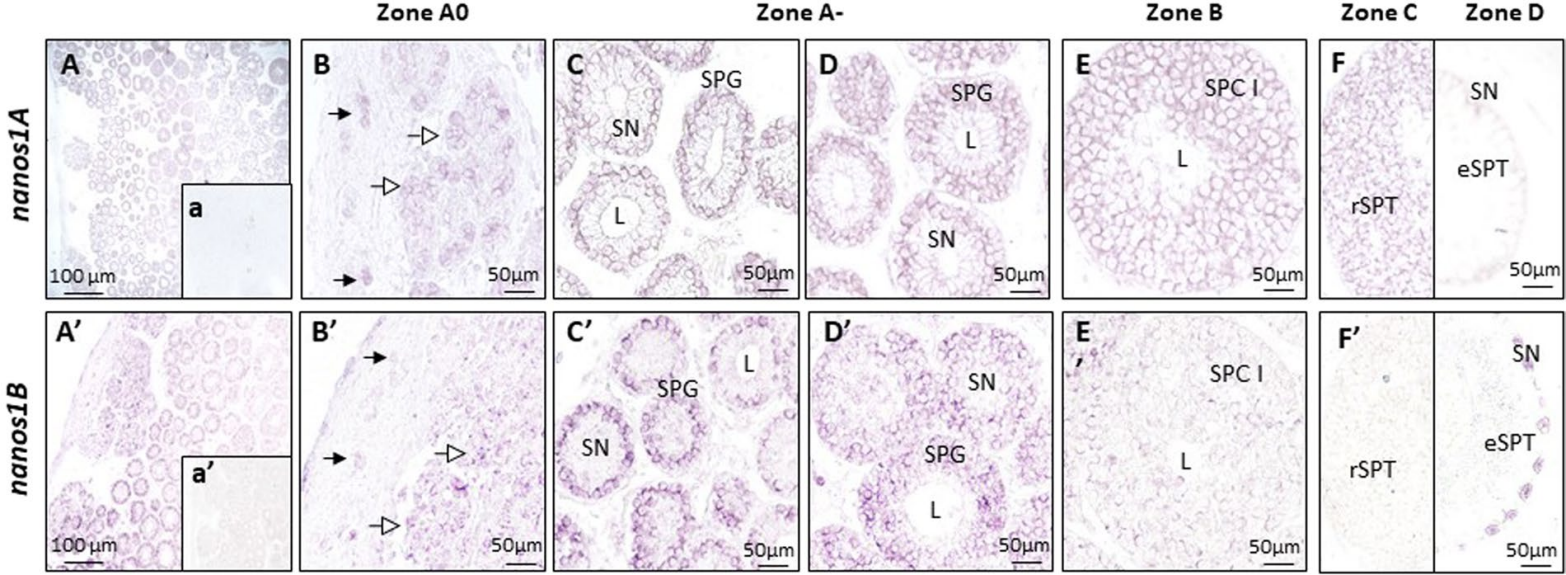
Different but both germ cell-specific expressions of *nanos1A* and *nanos1B* in dogfish testis. The cellular distribution of *nanos1* mRNAs was evaluated by *in situ* hybridization on dogfish testicular sections with antisense digoxigenin-conjugated riboprobes directed against *nanos1A* (**A**–**F**) or *nanos1B* transcripts (A’–F’). a and a’ represent sense riboprobes ISH for *nanos1A* and *nanos1B* respectively. The *nanos1A* transcripts were detected in the potential SSC (filled arrow) and in undifferentiated spermatogonia (open arrow) of the zone A0 (**B**), in the spermatogonia (SPG) of zone A- (**C**,**D**) as well as in the primary spermatocytes (SPCI) of the zone B (**E**) and in the round spermatids (rSPT) of the zone C (F left panel). No signal was observed in the elongated spermatids (eSPT) of the zone D (F right panel) nor in the Sertoli cells (their nuclei are indicated by SN). The *nanos1B* transcripts were mainly detected in the potential SSC (filled arrow), the undifferentiated spermatogonia (open arrow) of zone A0 (B’) and in spermatogonia (SPG) of zone A- (C’,D’). A marked decrease in *nanos1B* expression was observed in the primary spermatocytes (SPCI) of zone B (E’) and no transcript was present in the germ cells of zone C (F’ left panel) and zone D (F’ right panel). Aspecific labelling was observed in Sertoli cells nuclei of zone D using *nanos1B* riboprobes (F’ right panel). L: lumen of the cyst; A, A’: overview of zone A of testis.

Both *nanos1* transcripts were localized by *in situ* hybridization in testicular germ cells but with different patterns (Fig. 5). The transcript of *nanos 1* *A* showed a broad distribution in germ cells at all stages of differentiation (Fig. 5A–F). It was detected in potential SSCs isolated in a conjunctive tissue (filled arrow, Fig. 5B) in the A0 germinative zone, in undifferentiated spermatogonia (open arrow, Fig. 5B) in forming cysts and in differentiated spermatogonia (SPG) in cysts composed of one to four layers of spermatogonia with an adluminal crown of Sertoli cells nuclei (Fig. 5C,D). This *nanos1A* transcript was also detected in later stages, in zone B in spermatocytes (SPC, Fig. 5E), in zone C in round spermatids (rSPT, Fig. 5F left panel) but was absent in zone D in elongated spermatids (eSPT, Fig. 5F right panel). In contrast, *nanos1B* showed a strong expression restricted to spermatogonia (Fig. 5A’–F’). This transcript was detected from potential SSCs (filled arrowheads, Fig. 5B’) to differentiated spermatogonia (SPG, Fig. 5C’,D’). This expression was greatly diminished and almost undetectable in cysts containing primary spermatocytes (SPC I, Fig. 5E’) and undetectable at later stages in germ cells (Fig. 5F’). Sense riboprobes gave no signal (Fig. 5a,a’) except in Sertoli cells nuclei of some cysts in zone D using *nanos1B* sense riboprobes suggesting that the signal frequently observed in the nucleus of Sertoli cells in zone D with *nanos1B* antisense riboprobes would also be considered as unspecific (Fig. 5F’ right panel). So, both *nanos1* transcripts showed a germ cell-specific expression with a broad distribution to all stages of spermatogenesis for *nanos1A* and a spermatogonial-restricted expression pattern for *nanos1B*.

### In ovary, both *nanos1* mRNAs were detected in early previtellogenic oocytes and, later, in follicular cells surrounding vitellogenic oocytes

In female gonads, *nanos1A* and *nanos1B* showed similar expression patterns by *in situ* hybridization. Both transcripts were detected in primary oocytes of primordial follicles (Fig. 7A,A’). These oocytes are located near the ovary epithelium and are surrounded by some undifferentiated follicular cells which were not labelled (Fig. 7A,A’). This cytoplasmic expression progressively decreased in oocytes of previtellogenic follicles (Fig. 7B,B’). In previtellogenic follicles, follicular cells differentiate in two types: granulosa cells adjacent to zona pellucida and theca cells outside the basal lamina. Theca is vascularised and consists in fibroblastic-like inner cells and cuboidal outer cells. In vitellogenic follicles, *nanos1* transcripts were detected in both granulosa and theca cells (Fig. 7C,C’) whereas undifferentiated follicular cells previously showed no signal in primordial follicles (Fig. 7A,A’). In larger vitellogenic follicles which have multilayed granulosa cells, *nanos* transcripts were still present in both granulosa and theca cells (Fig. 7D,D’). As expected, sense riboprobes gave no signal (Fig. 7E,E’). So, *nanos1A* and *nanos1B* transcripts were both first detected in primary oocytes before vitellogenesis and later, in granulosa and theca cells surrounding vitellogenic oocytes.

**Figure 6:**
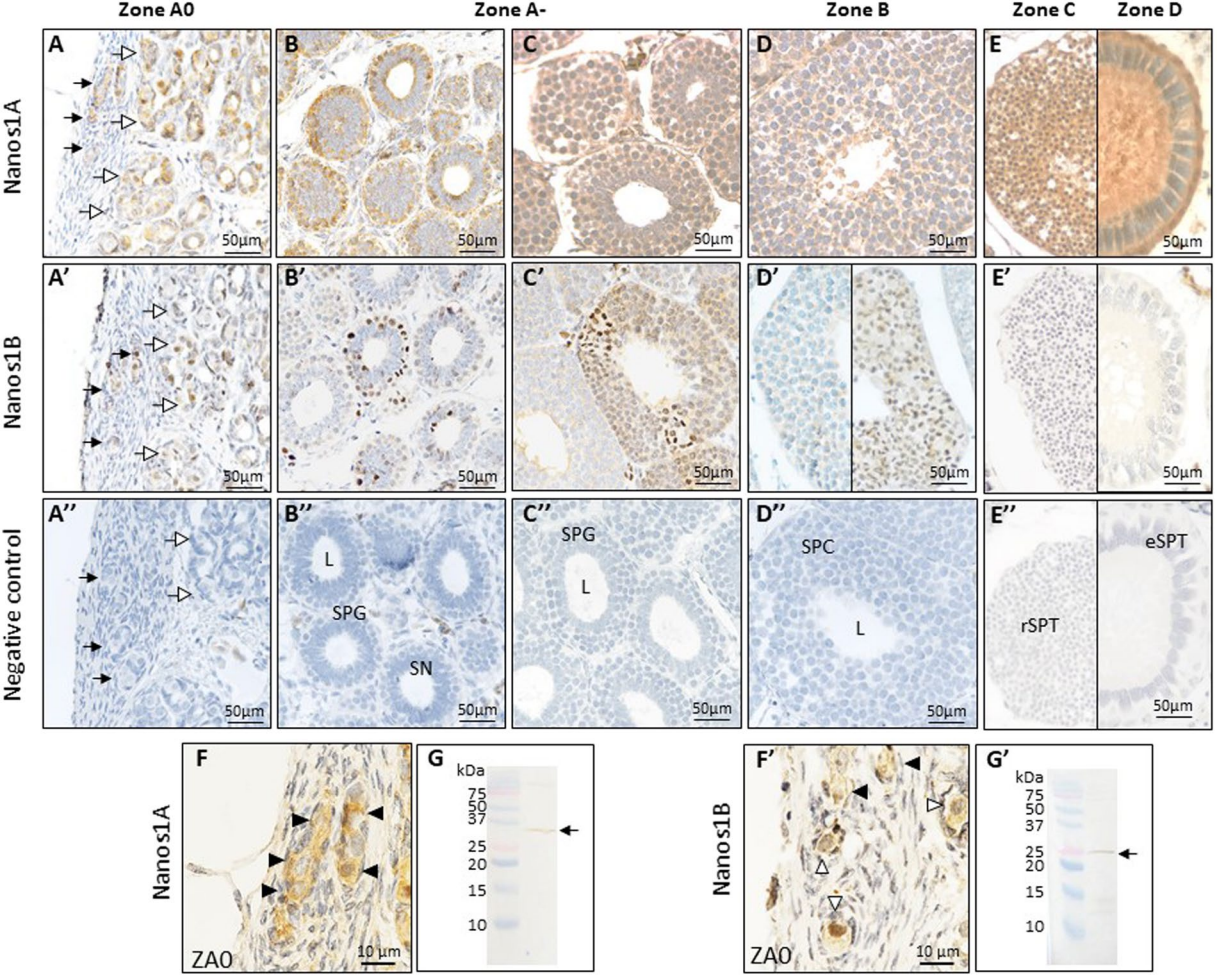
Different subcellular localizations of Nanos1A and Nanos1B proteins in dogfish testis. Immunohistochemistry has been performed on testis paraffin sections to detect Nanos1A (**A**–**F**) and Nanos1B (A’–F’). 3,3′-diaminobenzidine (DAB) has been used for the revelation. A”–E” represent the negative control. The Nanos1A protein was detected in potential SSC (A filled arrow) and in undifferentiated spermatogonia (open arrow) leaving zone A0 (**A**). In these cells, Nanos1A localized in the cytoplasm (F filled arrowhead) and in the nucleus (F open arrowhead). The protein was also detectable during the different stages of spermatogenesis but only in germ cell cytoplasm as observed in zone A (**B** and **C**) in spermatogonia (SPG), in zone B (**D**) in spermatocytes (SPC), in zone C (E left panel) in round spermatids (rSPT) and finally in zone D (E right panel) in elongated spermatids (eSPT). In contrast, Nanos1B was expressed in potential SSCs (filled arrow, A’), undifferentiated spermatogonia (open arrow, A’) leaving the germinal niche and a fraction of differentiated spermatogonia (B’,C’) and spermatocytes (D’ right panel). No expression was detected in zones C (**E** left panel) and D (E right panel). Specific nucleus expression was noticed in some potential SSCs (F’ open arrowhead) and a specific DNA labelling was detected in spermatogonia and spermatocytes in division (A’–D’). The specificity of the antibodies used was validated by Western-blot analysis (**G**,G’). Male brain protein extract was used as *nanos1* expression was the highest in this tissue. Proteins migrated in 15% SDS PAGE gels to separate low molecular weights since Nanos2 and Nanos3 theoretical molecular weights were estimated at 17.4 and 22.8 kDa respectively. Single bands were detected around the expected molecular weights, respectively 27.1 and 25 kDa for Nanos1A and Nanos1B.

**Figure 7:**
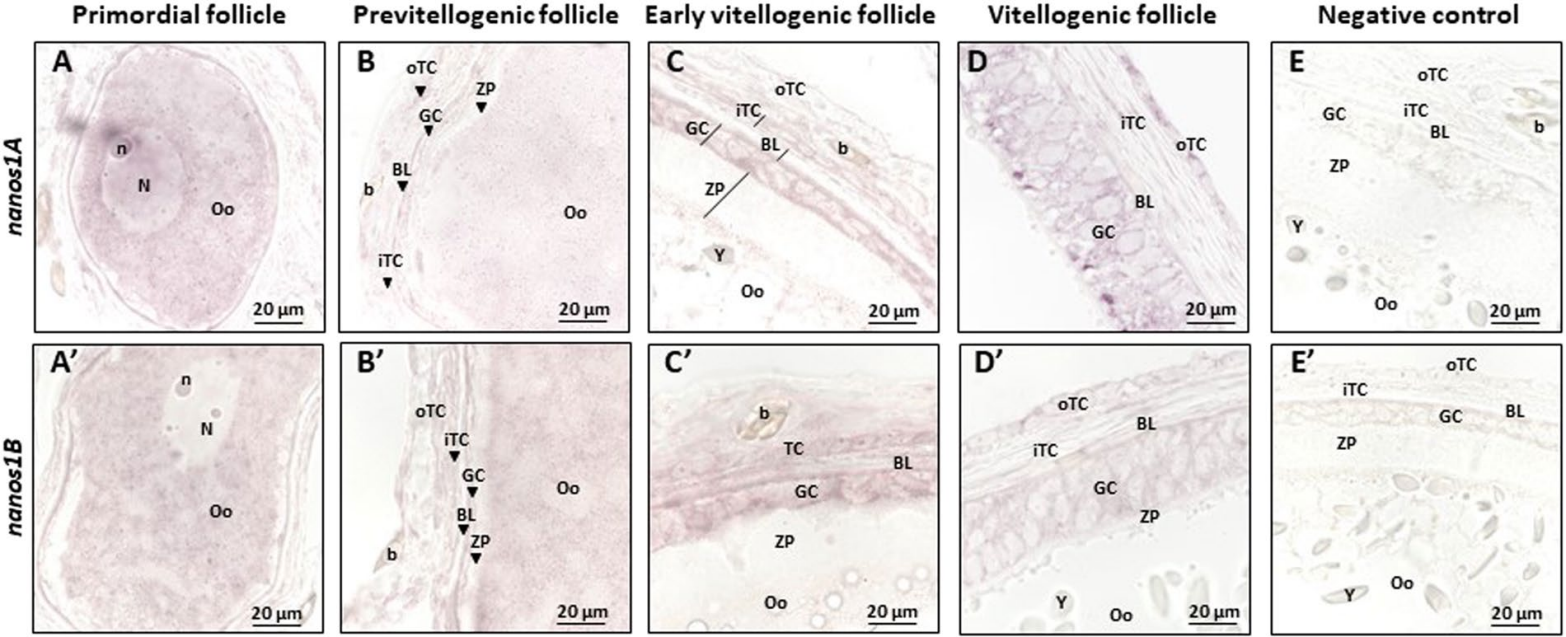
Similar distribution of *nanos1A* and *nanos1B* transcripts in germ cells and in follicular cells in dogfish ovary. The localization of *nanos1* mRNAs was determined in ovaries *by in situ* hybridization using antisense digoxigenin-conjugated riboprobes directed against *nanos1A* (**A–D**) or *nanos1B* transcripts (A’–D’). E and E’ represent results obtained with sense riboprobes for *nanos1A and nanos1B* respectively. The *nanos1A* transcripts were detected in primary oocyte of primordial follicles (around 100 µm in Ø illustrated in A). This labelling progressively decreased during oocyte growth until being undetectable in previtellogenic follicles (around 300 µm in Ø illustrated in (**B**). The mRNAs were also detected in the granulosa cells (GC) and the outer theca cells (oTC) of early (**C**) and the more advanced (**D**) vitellogenic follicles (around 500 µm and >2 mm in Ø respectively). The same expression profile was detected for *nanos1B* with an expression in primary oocyte of primordial follicles (A’), in granulosa (GC) and outer theca cells (oTC) of early (**C**) and more advanced (**D**) vitellogenic follicles. In previtellogenic follicle (B’), *nanos1B* was still detectable in oocyte cytoplasm. b: blood cells, BL: basal lamina, GC: granulosa cells, N: nucleus, n: nucleolus, Oo: primary oocyte, TC: theca cells, iTC: inner theca cells, oTC: outer theca cells, Y: yolk, ZP: zona pellucida.

### Nanos1 proteins showed distinct sub-cellular localizations in dogfish gonads

**Figure 8:**
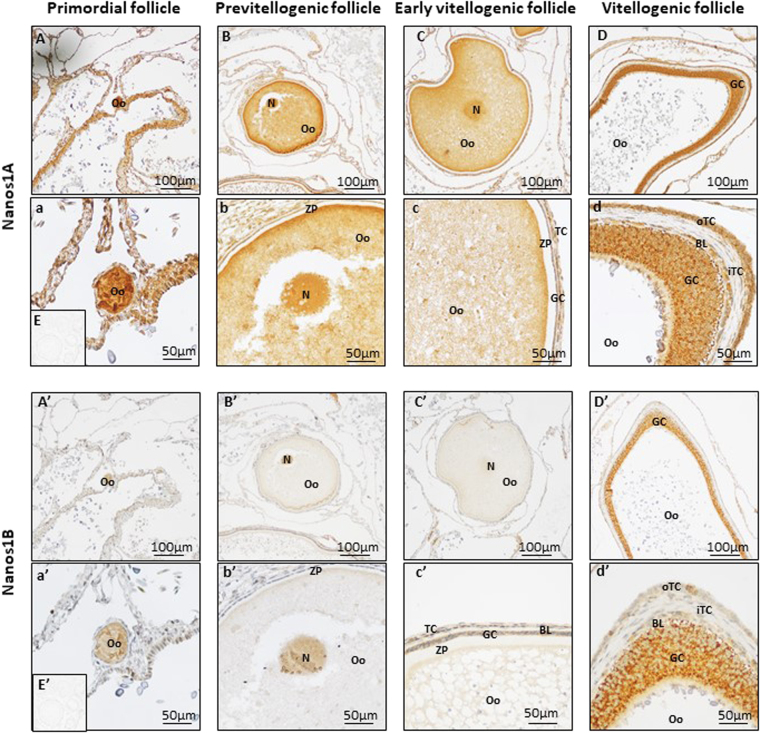
Nanos1A and Nanos1B proteins share a similar expression profile in dogfish ovary. Immunohistochemistry has been performed on dogfish ovaries paraffin sections to detect Nanos1A (**A**–**D**, higher magnification in a–d) and Nanos1B (A’–D’, higher magnification in a’–d’). 3,3′-diaminobenzidine (DAB) was used for the revelation. E and E’ illustrate the negative control. The Nanos1A protein was detected in primary oocytes from primordial follicles (around 100 µm in diameter, A,a) to early vitellogenic follicles (around 500 µm in Ø, **C**,c) in the cytoplasmic and nuclear (N) compartments. Staining in oocytes became progressively undetectable whereas it appeared in granulosa (GC) and outer theca cells (oTC) of early (**C**,c) and more advanced vitellogenic follicles (>2 mm in Ø, **D**,d). Similarly, Nanos1B was detected in oocytes of primordial follicles (A’,a’). In previtellogenic follicle (B’b’), Nanos1B presented a major expression in oocyte nucleus (N). Finally, Nanos1B was observed in granulosa cells (GC) and outer theca cells (oTC) in vitellogenic follicles (C’,c’ and D’,d’). BL: basal lamina, GC: granulosa cells, iTC: inner theca cells, N: nucleus, Oo: primary oocyte, oTC: outer theca cells, ZP: zona pellucida.

To localize Nanos1A and Nanos1B proteins in dogfish gonads, immunohistochemistry was performed on mature testes and ovaries using two primary antibodies (sc-366152 and ab174139) whose specificity was confirmed using Western-blots (Fig. 6G,G’). In testis of mature male dogfish, Nanos1A protein was detected in the cytoplasm of germ cells at all stages of spermatogenesis (Fig. 6A–E). This strong labelling was localized in the cytoplasm of potential SCCs (filled arrow, Fig. 6A–F) in the germinal niche (ZA0), in undifferentiated spermatogonia in cysts in formation (open arrow, Fig. 6A), in differentiated spermatogonia (SPG, Fig. 6B,C), in spermatocytes in zone B (SPC, Fig. 6D), in round spermatids in zone C (rSPT, Fig. 6E left panel) and in cysts with elongated spermatids in zone D (eSPT, Fig. 6E right panel). For this last one, labelling was associated to the lumen of the cyst, the middle piece and flagella of spermatids and also to the basal compartment of Sertoli cells (Fig. 6E right panel). In contrast, Nanos1B protein showed a restricted expression pattern limited to spermatogonia and primary spermatocytes. Cytoplasmic Nanos1B proteins were detected in most potential SSCs (Fig. 6A’,F’), in some undifferentiated spermatogonia (Fig. 6B’), in a lower proportion of differentiated spermatogonia (Fig 6B’,C’) and of primary spermatocytes (Fig. 6D’). This cytoplasmic labelling was associated to a nuclear labelling in some SSCs (Fig. 6F’) and undifferentiated spermatogonia (open arrow, Fig. 6A’). In differentiated spermatogonia and primary spermatocytes, a labelling associated to chromosomes was also observed in dividing cells (Fig. 6B’–D’). No signal was detected in spermatids in zones C and D (Fig. 6E’ left and right panels). In female, Nanos1 proteins were localized in primary oocytes in previtellogenic follicles and in follicular cells at later stages (Fig. 8). Primary oocytes presented cytoplasmic and nuclear Nanos1A protein in primordial follicles (Fig. 8A,a) and previtellogenic follicles (Fig. 8B,b). The intensity of the cytoplasmic labelling was decreased according to the growth of the oocyte. In addition, early and more advanced vitellogenic follicles displayed a strong staining in both granulosa and theca cells (Fig. 8C,d). In contrast, Nanos1B protein was detected in oocyte cytoplasm and nucleus of primordial follicles (Fig. 8A’,a’) and in oocyte nucleus of previtellogenic follicles (Fig. 8B’,b’). At this stage, labelling in oocyte cytoplasm was extremely weak and no follicular cell was stained. In vitellogenic follicles, Nanos1B expression was confirmed in the cytoplasm of granulosa cells and of outer theca cells but not in the inner theca cells (Fig. 8D’). So, Nanos1A and Nanos1B proteins showed cell-type specific distributions in accordance with their respective mRNAs in dogfish gonads, and, interestingly, a different cellular sub-localization since Nanos1B displayed a specific DNA association in early germ cells in male gonad. Our data suggest that Nanos1A and Nanos1B may have distinct functions.

## Discussion

In the present study, we first revealed the unexpected presence of two forms of *nanos1* in three Elasmobranch species (small-spotted dogfish, whale shark and little skate), both proteins contained the conserved Nanos family CCHC zinc finger motifs necessary for its function. This result led us to investigate *nanos1* gene copies origin and evolution using two complementary approaches, phylogenetic reconstructions and analyses of syntenic chromosomal fragments. Both *nanos1* paralogs identified in Chondrichthyes were also found in its sister group, the Osteichthyes, in ancient lineages of Actinopterygii (spotted gar) and of Sarcopterygii (coelacanth). In addition, paralogs of two genes of *nanos1A* synteny were localized in the vicinity of *nanos1B* in human and in teleostean fish. In contrast, *nanos1A* was the only *nanos1* gene copy found in an Agnatha genome (sea lamprey). Considering this, we hypothesize that the second round (2R) of whole genome duplication (WGD) that occurred after the divergence of Agnatha from Gnathostomes ancestor^27,28^, about 450 million years ago (MYA)^29,30^, gave rise to *nanos1B* gene copy. However, in contrast to drosophila *nanos* gene, *nanos1A* and *nanos1B* have no intron and we cannot exclude that they have emerged through a retroposition event from a cDNA. Synteny analyses showed that Tetrapods have lost the *nanos1B* gene copy. Teleosts underwent a third round (3 R) of whole genome duplication about 350 MYA^31,32^ which gave rise to *nanos1Ba* and *nanos1Bb* ohnologs and to the loss of *nanos1A* gene copy. The loss of gene copies can be explained by the rapid rediploidization process following genome duplication events. Two Teleost specific *nanos1* genes displaying different expression patterns were previously described in Acanthopterygians fish^11^. The synteny results support the previous hypothesis of a duplication of *nanos1B* in the ancestor at the base of the teleost radiation. Nanos1B proteins are highly conserved in their C-terminal zinc finger domain, binding targeted RNAs, but also outside this domain. The presence of protein partner interaction sites could explain this high pressure of selection. The particular segregation of Nanos1Ba and Nanos1Bb paralogs in the phylogenetic tree could be due to the high pressures of identical selective forces on both paralogs, inside the different Teleost families, to evolve in parallel and to conserve interactions with the same protein partners^33^. In comparison, Nanos1A protein sequences showed a much lower conservation at the N-terminal end in Vertebrates. Changes in the protein sequence may have created new opportunities for Nanos1A to acquire new functions in Tetrapods as a single gene copy is available in these species. Another gene involved in germline stemness, *pou5f1* (*oct4)*, was shown to result from the duplication of an ancestral *pou2* gene in Vertebrates. The paralogs *pou5f1* and *pou2* were respectively lost in Teleosts and in Mammals, and some Sarcopterygii displayed both copies^34^.

The tissue expression profiles of the two *nanos1A and nanos1B* paralogs described in this study in *Scyliorhinus canicula* are consistent with previous works on these paralogs in Tetrapods and Actinopterygii. Indeed, both paralogs are expressed *in the dogfish brain* as previously reported for Nanos1 in mouse, sturgeon and medaka^11,13,35^. However, the role of Nanos1 in brain remains unclear. In *nanos1* deficient mice, no significant neural abnormality was observed and no neural defect in term of behaviour has been identified^13^. A multiple tissue distribution was detected for *nanos1B*, an expression pattern also found for this gene copy for both *nanos1a* (*nanos1Ba* in the present study) and *nanos1b* (*nanos1Bb*) in two teleostean fishes, medaka^11^ and orange-spotted grouper^36^. A broad tissue distribution was also observed for Nanos1(B) in a chondrostean fish (Chinese sturgeon), which emerged after the Chondrichthyes but before the 3 R WGD of the Teleosts^35^.

Concerning *nanos* expression in gonads, most published studies focused on *nanos2* and *nanos3* paralogs which showed highest expression levels in testis in human^37^ and in testis and in ovary respectively in teleostean fishes^12,36^. However, *nanos1* was also revealed to be expressed in gonads in addition to brain in Tetrapods and Actinopterygii. In the shark *S. canicula*, *nanos1A* and *nanos1B* were both detected in ovary and testis. In male gonads, *nanos1* paralogs displayed different expression patterns. Transcripts of *nanos1A* were found in all testicular regions except zone D by real-time PCR. This result was confirmed by *in situ* hybridization with a germinal localization of mRNAs. By immunohistochemistry, Nanos1A protein showed a cytoplasmic distribution in germ cells at all stages. Human and rodents also present a large distribution of NANOS1 protein in germ cell cytoplasm^38^ and in nuage structures like the chromatoid body^39^. NANOS1 was also strongly detected in residual bodies of spermatids in rat^40^. This could explain the diffuse signal obtained in cysts containing elongated spermatids and during the spermiation process associated to the progressive degeneration of Sertoli cells in the dogfish^41^. Concerning its potential function, mutations of *nanos1* in human induced a Sertoli-cell-only syndrome resulting from a complete lack of germline cells in the seminiferous tubules^14^. This observation supports an important role of this gene in germ cell lineage maintenance. In contrast to the large distribution of *nanos1A* in testis, dogfish *nanos1B* was expressed in the germinative zone and in zone A- containing cysts with spermatogonia but rapidly decreased at later stages of spermatogenesis. In accordance with this RT-PCR result, this transcript was strongly detected from the potential SSCs to differentiated spermatogonia, but was almost undetectable in spermatocytes and was not detected in cysts of spermatids. The different expression patterns of *nanos1* genes in testis could be due to different epigenetic regulations, or to an evolution of the promoter region of *nanos1* gene correlated with different regulatory transcription factors. The stability of the transcripts can also be differentially regulated, for example through specific miRNA. Few descriptions of Nanos1 are available in Actinopterygii. In the primitive chondrostean fish, *Acipenser sinensis*, Nanos1 was shown to be expressed in germ cell cytoplasm in 4.5 year-old individuals but was not assessed in adult sturgeons^35^. Surprisingly, the teleostean fish medaka presented an expression of *nanos1(B)a* in somatic cells enclosing spermatocytes and spermatids and no expression of *nanos1(B)b* was observed in testis^11^. More studies on its expression and function in the testes of Actinopterygii are needed to understand the evolution of the *nanos1* paralogs in Vertebrates.

In dogfish ovary, *nanos1A* and *nanos1B* transcripts and proteins were both detected in oocytes in primordial follicles. This expression progressively decreased during oocyte growth in previtellogenic oocytes and was no more detectable in vitellogenic oocytes. Granulosa cells and outer theca cells surrounding vitellogenic oocytes expressed *nanos1* paralogs contrary to follicular cells at earlier stages. Nanos1 proteins were detected in the cytoplasm but also in the nucleus of primary oocytes, particularly strongly at previtellogenic stage. Contrasting results were reported concerning *nanos1* expression in the ovary depending on the species. *Nanos1* was not detected in ovary in human^38^ like *nanos1b* in medaka^11^. Oocytes but not follicular cells were shown to present *Nanos1* expression in juvenile sturgeon^35^, in mouse (by LacZ reporter system under *Nanos1* promoter region^13^), and in *Xenopus*. More precisely, *Nanos1* mRNA (*Xcat2*) was concentrated in germ plasm components of early staged oocytes in frog without translation before fertilization^42–44^. In contrast, *nanos1a* was localized in somatic cells surrounding oocytes in female medaka at 10 days post-hatching but was undetectable in adult ovary^11^. Interestingly, Vasa, another maternal factor specifically expressed in germ cells, was described in germline cells but also in somatic cells in the ovary of adult drosophila and lizard. This protein was indeed localised in the nurse cells throughout early stages of oogenesis in drosophila^45^. In the lizard, Vasa protein was detected in intermediate and pyriform follicular cells surrounding the oocyte in immature follicles^46^. In both species, Vasa protein is transferred to the oocyte cytoplasm at later stages of oogenesis. In summary, no consensual expression pattern could be determined for *nanos1* in the ovary of Vertebrates but both the oocyte and follicular cell expressions found in dogfish were already reported in other species and we cannot exclude the transfer of proteins between somatic and germinal cells as it was described for Vasa protein.

Nanos1B was detected in the nucleus of potential SSCs and co-localized with chromosomes during mitosis of spermatogonia and meiosis of primary spermatocytes in dogfish testis and in the nucleus of early primary oocytes in ovary. A co-localization of a Nanos protein with chromosomes during mitosis and meiosis has been previously reported in adult human germ cells for NANOS3 but not for NANOS1^37^. NANOS3 protein showed the highest expression in the nucleus of oocytes in primordial follicles and in oocyte cytoplasm until antral follicles in human ovary and was detected in the nucleus of male germ cells and co-localized with chromosomes during cellular division in testis. In addition, during foetal life, NANOS1 was detected in gonocyte nuclei while during post-natal life, it was distributed in nucleus and in perinuclear cytoplasm in human and marmoset gonocytes^47^. This raises the question of the evolution of the functions of these proteins and their potential role during mitosis/meiosis.

Nanos was shown to play a crucial role in the maintenance of germline stem cells, through the suppression of meiosis in fly^6^, nematode^48^, mice^9^ and zebrafish^49^. More precisely, the conserved carboxy terminal region of this RNA-binding protein binds the 3′ untranslated region of mRNAs to repress their translation^2^, in association with other proteins such as Pumilio (for review^50^). However, in Vertebrates, the different paralogs of *nanos* play distinct roles in germ cell fate but also functional redundancy. For instance, Nanos3-null female are infertile but Nanos2 expression in these females rescues their defects^51^. Our study supports a role of Nanos1A and Nanos1B in dogfish male and female germ cells. Although their expression patterns were similar in the ovary, they showed a partial overlapping distribution in the testis, suggesting a possible redundancy but also most probably distinct roles for these two Nanos1 paralogs.

To conclude, this study sheds light on the origin and the evolutionary history of two distinct *nanos1* paralogs in Vertebrates. Our observations revealed a hidden paralogy resulting from an ancient duplication of the *nanos1* gene at the basis of Vertebrates and different gene copies losses between Tetrapods and Teleosts. Both gene copies showed different evolutionary scenarios since, in contrast to *nanos1A*, *nanos1B* was maintained and duplicated in the Teleosts and the amino acids sequence was highly conserved for hundred million years in fish species only. In dogfish, a basal Gnathostome, we demonstrated that *nanos1A* and *nanos1B* showed different expression patterns in gonads consistent with possible distinct roles in germ cell fate regulation. However, further investigations will be required to understand the biological significance of the coexistence of four *nanos* paralogs (*nanos1A, nanos1B, nanos2, nanos3*) in basal Vertebrates.

## Materials and Methods

### Phylogenetic and synteny analyses

Nanos sequences were selected using the TBLASTN algorithm and mouse NANOS protein sequences as queries to identify homologous sequences in ESTs, mRNAs and genome databases deposited at the NCBI or EMBL institutes. Dogfish *nanos1* transcripts were found in a library built from embryos, juveniles and adult tissues^21,22^ and in dogfish gonadal transcripts libraries generated by Illumina (Phylofish ANR funded project coordinated by J. Bobe and Y. Guiguen, Fish Physiology and Genomics unit, Rennes, France). In addition, dogfish *nanos* genes were identified and localized in dogfish genome (GenoShark project). Dogfish *nanos* sequences were submitted to Genbank. Sequences accession numbers are detailed in Supplementary Table S5. Nanos protein sequences were aligned using BioEdit ClustalW multiple alignment editor software version 7.1.3.0 (http://www.mbio.ncsu.edu/BioEdit/bioedit.html) and phylogenetic trees were built using the Molecular Evolutionary Genetics Analysis (MEGA) software version 7.0^52^. Trees were constructed using the Neighbour-Joining method and the reliability of the inferred trees was assessed using the bootstrap procedure with 1000 replications.

Nanos1 protein sequences representative for Sarcopterygii (coelacanth, chicken and human), Actinopterygii (spotted gar, arowana and salmon) and Chondrichthyes (dogfish and chimera) were aligned using BioEdit software. Conserved Cys-Cys-His-Cys zinc finger motifs, essential for Nanos function, were annotated^2^.

The *nanos* genes were mapped on the genome of different vertebrate species using NCBI or Ensembl genome browsers. Neighbouring genes that were not annotated were determined using the amino acid sequence of heterologous counterparts as query sequence. The annotation of the newly detected genes was validated using reciprocal blast queries.

### Animals

Adult male and female dogfish *Scyliorhinus.canicula* were captured from the English Channel using the facilities of the Lycée Maritime et Aquacole (Cherbourg, France). The aquarium La Cité de la Mer (Cherbourg) first stored them in natural seawater tanks waiting their transfer to the Centre de Recherches en Environnement Côtier (CREC, Luc sur Mer, France). The CREC facilities were approved by the direction of the Council Department of populations care (Direction départementale de la protection des populations du Calvados, préfecture du Calvados) under number A14384001. No specific permits were required for these field studies and procedures were performed according to the European directive 2010/63/UE for care and use of animals. The dogfish *S. canicula* is not an endangered or protected species according to the International Union for Conservation of Nature. Sharks were allowed to acclimate for at least 2 weeks before tissue sampling and were euthanized by percussive blow to the head followed by sectioning of the spinal cord and pithing (except when brain was collected). All efforts were made to minimize suffering. The personnel were trained and qualified for animal experimentation. Testes were sampled and transferred directly into Gautron buffer^24^ complemented by 58 mM trimethylamine-*N*-oxide (TMAO) and cut transversally into 2-mm slices fresh testicular sections. These sections were then dissected under a stereomicroscope into five zones (A0, A-, B, C, and D) based on their transillumination appearance as described previously^24^. All tissues were frozen in liquid nitrogen for RNA extraction or fixed 24 h by 4% paraformaldehyde (w/v in PBS) for immunohistochemistry and *in situ* hybridization.

### RNA extraction and purification

In RNase-free conditions, each frozen sample was grounded in 1 ml TRI-Reagent® by Ultra-Turrax® (IKA®T10 basic, Staufen, Germany). Total mRNA was extracted by complementation of 200 μl chloroform, homogenized and centrifuged for 15 min at 4 °C at 12 000 rpm. Aqueous phases were transferred into new tubes with 500 μl isopropanol (1:1, v/v), centrifuged, and pellets were rinsed twice with 75% ethanol and 100% ethanol (v/v) and air-dried at RT. Total RNAs were re-suspended in DEPC-treated water and quantified with a Nanodrop 2000 spectrophotometer (Thermo Scientific, Les Ulis Courtaboeuf, France).

### Reverse Transcription (RT)

To prevent any DNA contamination, 0.5 μg of total RNA was treated with 3 U RQ1 DNase (Promega, Madison, USA) in a 10 μl volume and incubated for 30 min at 37 °C. To stop the enzyme reaction 1 µl of Stop solution was added followed by 10 min heating at 65 °C. One µg random hexamers was added and total RNA was denatured for 5 min at 70  °C. The RT was immediately carried out for 1 h at 37  °C with 500 nM dNTP, 24 U RNasin, 200 U M-MLV-RT in 1x M-MLV RT buffer (Promega) in a 25 μl final volume.

### Real-time PCR

Real-time PCR was performed on an iCycler iQ apparatus (Bio-Rad, Hercules, USA) in duplicate and repeated using cDNA from three different animals for male and female. Panels of tissues included gills, brain, liver, muscle, fin, heart, spleen, epigonal tissue and eye. It was completed with shell gland, immature ovaries (containing previtellogenic oocytes) from sub-adult females and mature ovaries (containing also vitellogenic oocytes) from adult females, and epididymis and the testicular A0 zone, corresponding to the germinative area, in males. In addition, cDNA from four animals were used to compare mRNAs expression in the five testicular zones (A0, A-, B, C and D). RT-PCR primers were designed on *Nanos1a* and *Nanos1b* divergent parts of the nucleic sequence (Supplementary Table S6). Real-time PCR mix is composed 0.3 µM of each primer and 1x GoTaq® qPCR Master mix (Promega) in nuclease free water. In each well, 5 μl of 1:20 (for mRNA expression) and 1:2000 (for 5S rRNA normalization) diluted cDNA was added to the 20 µl of qPCR mix. The PCR cycle parameters were as followed: 1 × (95 °C, 2 min); 45 × [(95 °C, 30 s) and (60 °C, 45 s)]; and 80 × (55 + 0.5 °C, 10 s). Results were established with the iCycler Software (IQ^TH^ 3.1 Bio-Rad). For each plate, efficiency of PCR was assessed using appropriate dilution series, and single amplicon formation was confirmed on melting curves or validated by electrophoresis migration. The relative expression levels of *nanos1A* and *nanos1B* were normalized with the *5S rRNA*^53^. Data are presented as mean + standard deviation (SD). Differences in gene expression were evaluated using one-way analysis of variance (one-way ANOVA) followed up with Games-Howell test using Minitab software (Minitab 18 Statistical Software, Pennsylvania, USA). No significant difference was found in female tissues in comparison to the immature ovary. Asterisks (*) indicate values significantly different (p ≤ 0.05) to the testicular zone A0 in male tissues. Using the same statistical test, differences in gene expression between each different testicular zone were detailed. Testicular zones sharing no letter in common show significantly different expression levels with a p-value inferior to 0.05.

### *In situ* Hybridization (ISH)

Dig-conjugated riboprobes were synthesized from cDNA clones constructed in the pSPORT1 vector. Complementary DNAs were amplified through a standard PCR procedure with M13 forward and reverse primers (Supplementary Table S6). *In vitro* transcription was carried out on 1 μg purified PCR product using the DIG RNA labelling kit and in accordance to the manufacturer instructions (Roche, Mannheim, Germany). Riboprobes were purified by precipitation, ethanol washed and quantified using a Nanodrop 2000 spectrophotometer (Thermo Scientific, Washington, Delaware USA). The cDNA clones were built using the Superscript plasmid system with Gateway technology (Invitrogen), so that T7 and Sp6 enzymes produced respectively antisense and sense riboprobes.

Testicular cross sections, 0.5  cm-thick, and ovary samples were fixed in ice-cold 4% paraformaldehyde (w/v in PBS) for 24 h and progressively dehydrated in a series of PBS/ethanol solutions. Testis samples were incubated in butanol overnight and embedded in paraffin to be stored at 4 °C before use. Five μm-thick cross sections were cut with a microtome and mounted on Superfrost slides. Sections were deparaffinized by two bathes of Roti®-Histol, and rehydrated with ethanol bathes at decreasing concentrations (100%, 96% and 70% in DEPC water) and one PBS bath. Sections were then fixed in 4% PFA for 10 min, rinsed in PBS and permeabilized with 5 μg/ml proteinase K (Roche, Mannheim, Germany) in 0.05 M Tris and 0.01 M EDTA, pH 8. After two PBS washes, sections were refixed in 4% PFA for 10 min and washed with PBS. Sections were put in NaCl 150 mM for 5 min and then incubated for 1 h in prehybridization mix (deionised formamide 50% (v/v), 2X SSC, 5 mM EDTA pH8, 0.1% Tween 20, 1X Denhardt’s solution, 169 µM heparin, 0.1% CHAPS, 50 µg/ml tRNA) at 65 °C in a humid chamber. Hybridization was performed overnight in prehybridization mix containing 4ng/µl riboprobes. Sections were rinsed in SSC 1X and SSC 1.5X for 10 min at 65 °C and incubated in SSC 2X for 20 min twice at 37 °C. RNase A treatment (0.2 µg/ml in SSC 2X) was then performed for 30 min at 37 °C followed by one bath of SSC 2x at RT and 2 bathes of 30 min in SSC 0.2X at 57 °C. Sections were then incubated in 1x MABT (0.1 M maleic acid, 150 mM NaCl, and 0.1% Tween 20 in DEPC-treated water, pH 7.5) for 15 min twice and in a blocking solution (5% decomplemented sheep serum in 1x MABT) for 3 h at RT. Immunodetection was performed with an anti-DIG antibody coupled with alkaline phosphatase (1:2000 dilution, Roche, Mannheim, Germany) in blocking solution overnight at 4 °C. Several MABT 1X washes from 5 to 30 min were performed and pre-revelation treatment was done by 2X 15 min NTMT solution (100 mM NaCl, 100 mM Tris, pH 9.5, 50 mM MgCl_2_, and 0.1% Tween 20 in DEPC-treated water). Finally, staining was revealed by the addition of 61 µM NBT/57 µM BCIP (Roche, Mannheim, Germany) in NTMT solution until staining could be visually detected. Sections were mounted with Vectashield mounting medium. Images were acquired using a Nikon eclipse 80i microscope equipped with Nikon NIS-Elements D 3.0 Software (Nikon Instruments, Japan). Testicular sections were described according previously determined cysts stages^54^. Ovary sections were characterised based on histological studies in rays^55,56^.

### Immunohistochemistry

Dogfish testis and ovaries cross sections were cut from paraffin blocks and placed on polysine-coated slides. These 3-µm sections were deparaffinized in Roti®-Histol baths and rehydrated by a series of ethanol dilutions. Antigen unmasking was performed by a microwaves heating followed by a cooling for 1 h at room temperature (RT). Endogenous peroxidase activity was blocked by a bath of 30 min in 3% hydrogen peroxide /PBS, and the saturation of unspecific sites was performed by a 30 min incubation in 1% BSA (w/v) /triton 0.1% (v/v) /phosphate-buffered saline (PBS). Heterologous primary antibodies were used; a rabbit polyclonal primary antibody (1:50 dilution, sc-366152, Santa Cruz Biotechnology) produced against the conserved C-terminal sequence (amino-acids 205–250) of human Nanos1, closer to dogfish Nanos1A. The second rabbit polyclonal antibody used (1:50 dilution, ab174139, Abcam) was directed against the internal sequence (amino-acids 57–85) of human Nanos2, a peptide closer to Nanos1B as determined by blast against dogfish cDNA banks. Both were diluted in blocking solution and incubated overnight at 4 °C. Sections were rinsed and incubated for 2 h at RT with the secondary swine polyclonal antibody anti-rabbit immunoglobulins conjugated to HRP (1:200 dilution, PO399, Dako, Glostrup, Denmark). The revelation was performed by diaminobenzidine (DAB). After washes and counterstaining for 1 min in hematoxylin, sections were dehydrated and mounted in Roti-Histokit medium. Images were made using a Nikon Eclipse 80i microscope equipped with NIS-Elements D 3.0 software (Nikon Instruments, Japan).

### Western Blot

Proteins were extracted from male dogfish brain. Fresh tissue was homogenized on ice using conical pestles in 1 ml of lysis buffer (20 mM HEPES, pH 7.5; 1 mM EDTA; 0.5 mM DTT) and anti-protease mixture (1 mM AEBSF, 10 µM E64). Sample was sonicated three times for 5 s each with a pause of 30 s between each pulse and then centrifuged for 30 min at 14 000 rpm at 4 °C. Proteins (100 μg) from the supernatant were separated in 15% SDS PAGE gel and transferred to polyvinylidene difluoride membranes. After a blocking step of 1 h in PBS/3% BSA at room temperature, membranes were incubated overnight in the previously used primary antibodies diluted in blocking buffer (1:200). After five washes of 5 min each, membranes were incubated for 1 h 30 min at RT with the secondary swine polyclonal anti-rabbit IgG HRP-conjugated antibody (1:1000 dilution, P0399, DAKO). After washes, peroxidase reaction was developed with DAB solution (D4293; Sigma).

### Data availability

All data generated or analysed during this study are included in this published article (and its Supplementary Information files).

### Electronic supplementary material

Supplementary data
