# Author Correction: The *nanos1* gene was duplicated in early Vertebrates and the two paralogs show different gonadal expression profiles in a shark

**DOI:** 10.1038/s41598-024-56271-3

**Published:** 2024-03-18

**Authors:** Laura Gribouval, Pascal Sourdaine, Jean-Jacques Lareyre, Johanna Bellaiche, Florence Le Gac, Sylvie Mazan, Cécile Guiardiere, Pierrïck Auvray, Aude Gautier

**Affiliations:** 1 Normandie University, UNICAEN, Sorbonne Universités MNHN, UPMC University Paris 06, UA, CNRS, IRD, Biologie des Organismes et Ecosystèmes Aquatiques (BOREA), CS14032, 14032 CAEN Cedex 5, France; 2 KELIA, Parc Technopolitain Atalante Saint Malo, 35400 Saint Malo, France; 3 INRA UPR1037, Laboratory of Fish Physiology and Genomics, BIOSIT, Ouest-Genopole, Campus de Beaulieu, 35042 Rennes, France; 4 CNRS-UPMC-Sorbonne Universités, UMR 7232, Observatoire océanologique, 66650 Banyuls sur mer, France

Correction to: *Scientific Reports* 10.1038/s41598-018-24643-1, published online 02 May 2018

This Article contains an error in Figure 1, where “Chr 19” for the human chromosome carrying *nanos1A* should read “Chr 10”. The correct Figure 1 and the accompanying legend appear below.

**Figure 1:**
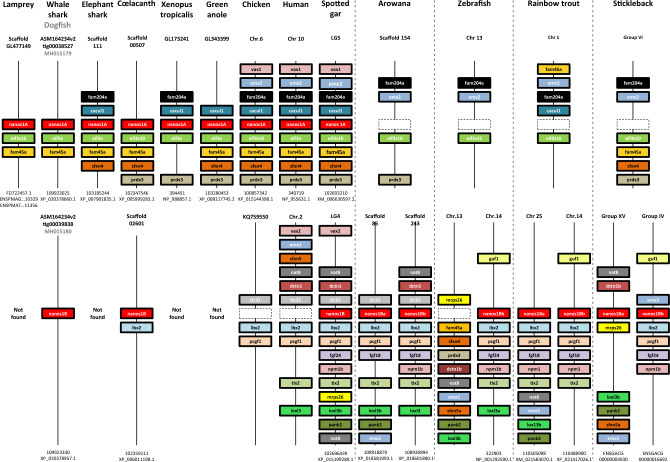
Gene synteny comparisons provide evidences that multiple *nanos1* gene duplications and losses occurred in vertebrates Three *nanos1* paralogs and their neighbouring genes showed syntenic genomic locations through Vertebrates. In the top panel, genes in the vicinity of a first copy, named *nanos1A*, were mapped. The figure was not drawn to scale. Each gene was represented by a specific coloured box. The name of each scaffold or chromosome harbouring the synteny is indicated at the top for each species whereas *nanos1* gene copy and protein accession numbers are detailed at the bottom. Although the structure of the chromosomal fragment was well conserved during the evolution, the *nanos1A* gene copy is not observed in the genome of Teleost fish as symbolized by the dotted boxes. In the bottom panel, the genomic environment of the second *nanos1* gene copy, termed *nanos1B*, was similarly represented. This different syntenic chromosomal fragment was not found in lamprey suggesting its apparition in Gnathostomata. Both *nanos1* paralogs (*nanos1A* and *nanos1B*) were detected In Chondrichthyes (dogfish and whale shark) and in Osteichthyes, respectively at the basis of Sarcopterygii (coelacanth) and of Actinopterygii (spotted gar). In contrast, *nanos1B* gene was not found in elephant shark, xenopus, green anole, chicken and human, suggesting its loss in these species. Teleosts showed two *nanos1B* gene copies carried by similar but distinct chromosomal fragments. The two *nanos1* paralogs in Teleosts were re-named *nanos1Ba* and *nanos1Bb*. Note that zebrafish is an atypical fish species because its genome does not harbour *the nanos1Ba* gene copy as indicated by a spotted box.

