# Supplementary material for: The nanos1 gene was duplicated in early Vertebrates and the two paralogs show different gonadal expression profiles in a shark
Source: Sci Rep. 2018 May 2;8:6942. doi: 10.1038/s41598-018-24643-1 (PMC5932020; doi:10.1038/s41598-018-24643-1)
Supplement: Supplementary file 1 — Supplementary data [file 41598_2018_24643_MOESM1_ESM.pdf]

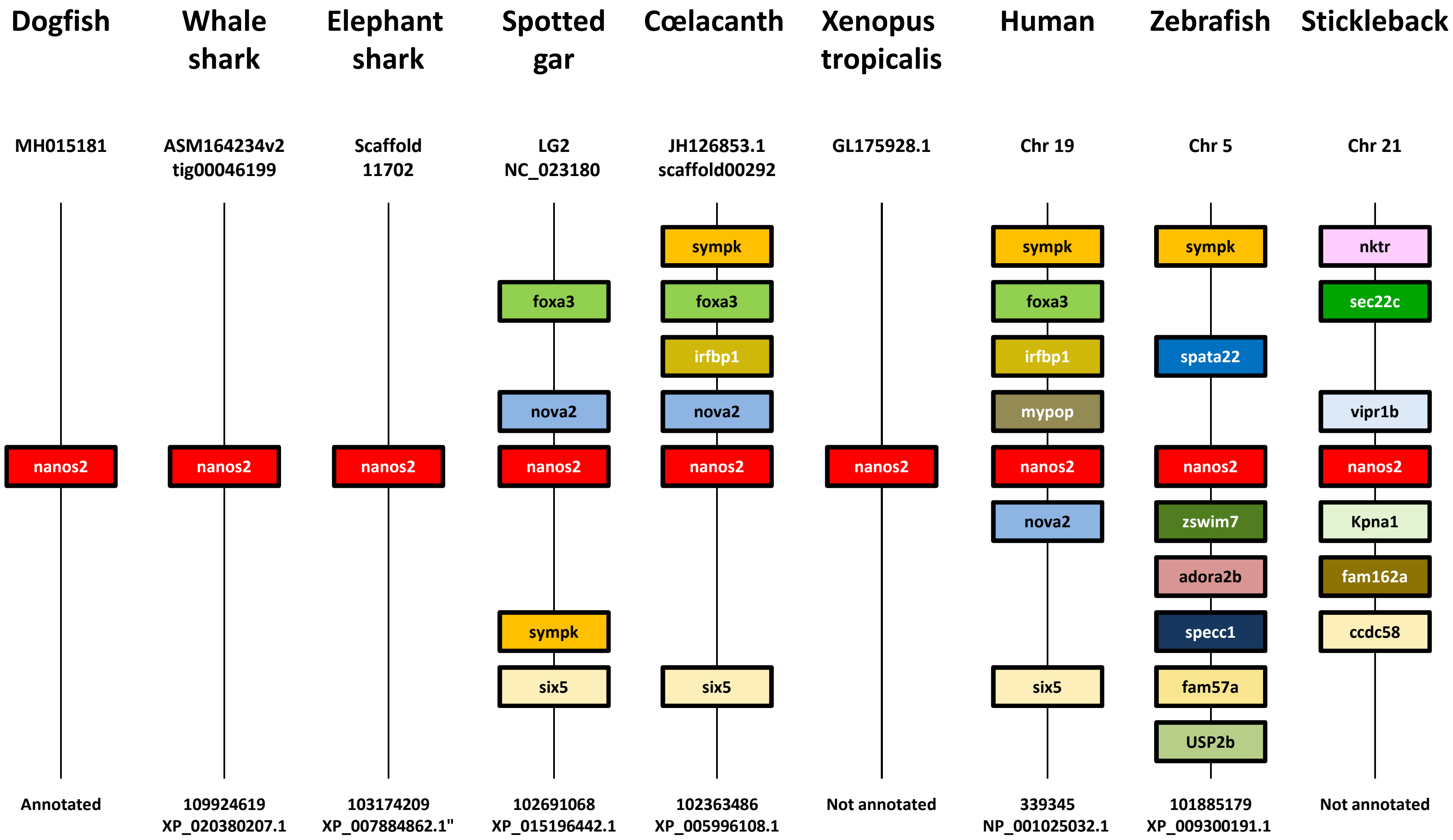

Supplementary Figure S1: Genomic location of *nanos2* gene in Vertebrate species.

The *nanos2* orthologs and their neighbouring genes, illustrated in specific coloured boxes, were mapped. Vertebrate representative species were chosen among Agnatha, Chondrichthyes and Osteichthyes. The figure was not drawn to scale. The name of each scaffold or chromosome harbouring the synteny is indicated at the top for each species whereas gene and protein accession numbers are detailed at the bottom. Concerning dogfish, whale shark, elephant shark and *xenopus tropicalis*, *nanos2* was localized on a too short chromosomic fragment to identify neighbouring genes. Gene synteny was well conserved between human, coelacanth and spotted gar but only *sympk* gene was found in the vicinity of *nanos2* in zebrafish. Note that the synteny appears disrupted in stickleback.

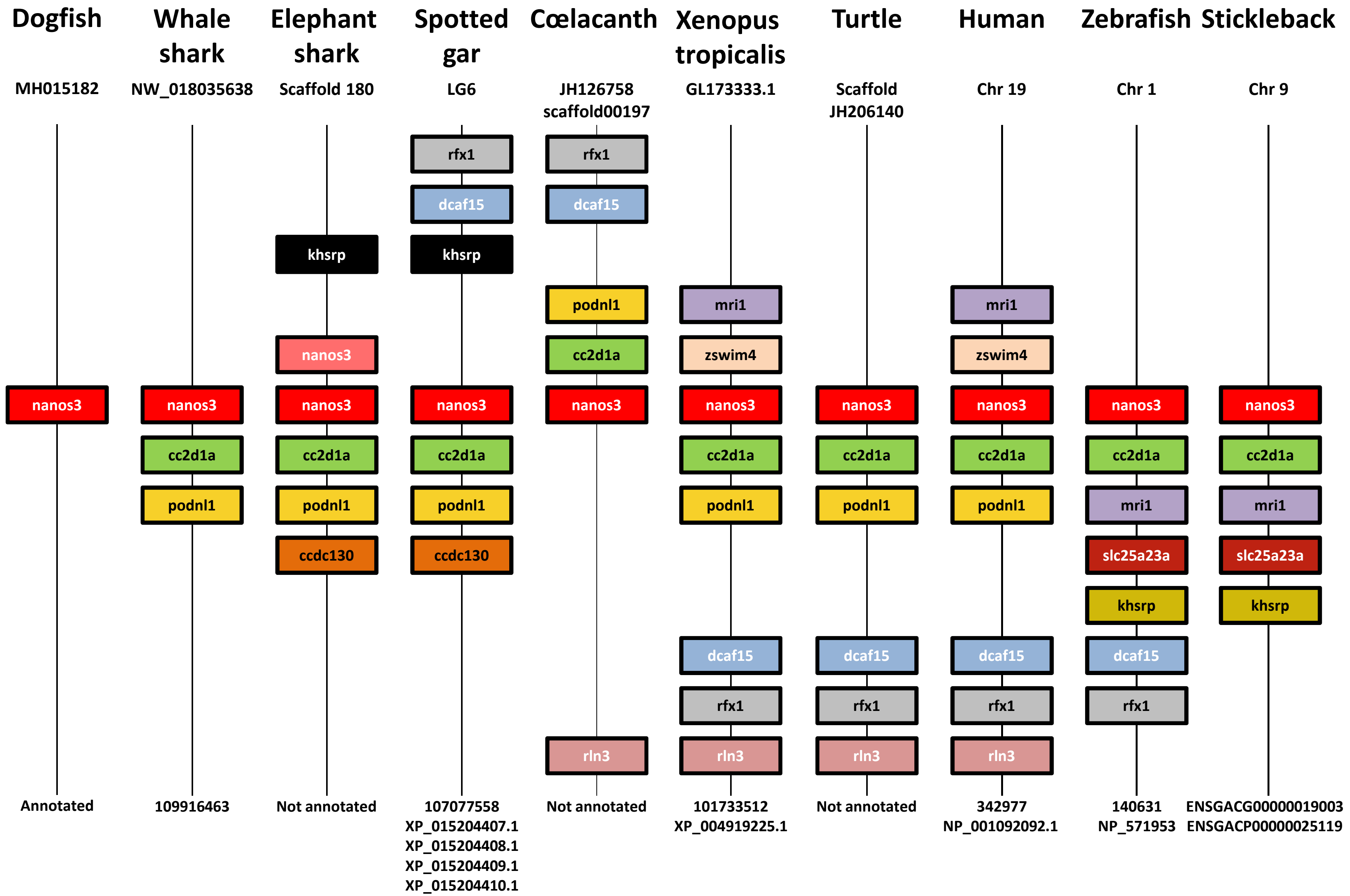

Supplementary Figure S2: Syntenic genomic location of *nanos3* gene in Gnathostomes.

The *nanos3* orthologs and their neighbouring genes, illustrated in coloured boxes, were mapped. Vertebrate representative species were chosen among Agnatha, Chondrichthyes and Osteichthyes. The figure was not drawn to scale. The name of each scaffold or chromosome harbouring the synteny is indicated at the top for each species whereas gene and protein accession numbers are detailed at the bottom. Gene synteny was well conserved from Chondrichthyes (whale shark and elephant shark) to Osteichthyes both in Sarcopterygii (coelacanth, xenopus, turtle and human) and Actinopterygii (spotted gar, zebrafish and stickleback). In dogfish, *nanos3* was localized in a too short chromosomic fragment to identify neighbouring genes.

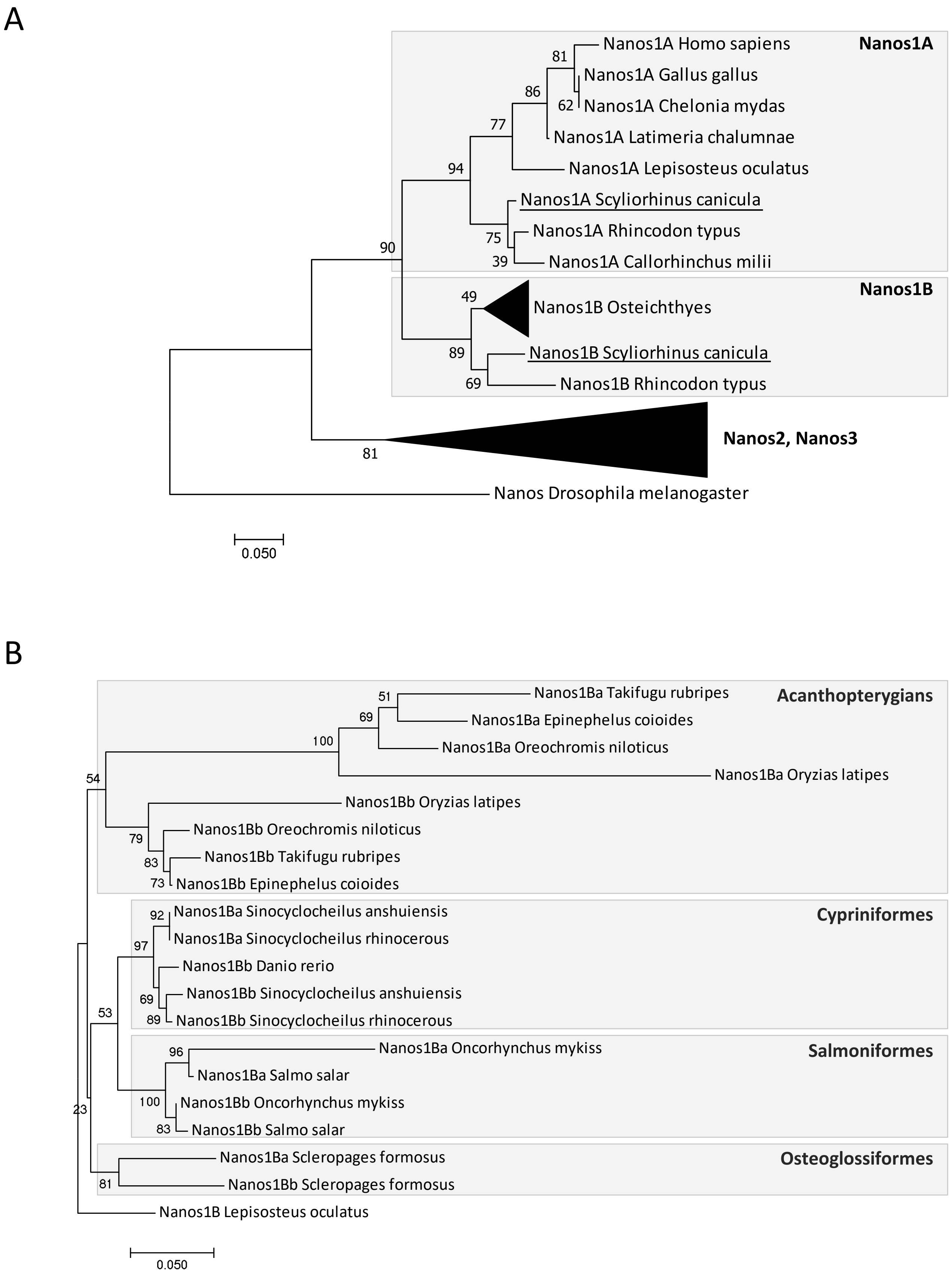

A
Nanos1A
Nanos1A Homo sapiens
Nanos1A Gallus gallus
Nanos1A Chelonia mydas
Nanos1A Latimeria chalumnae
Nanos1A Lepisosteus oculatus
Nanos1A Scyliorhinus canicula
Nanos1A Rhincodon typus
Nanos1A Callorhinchus milii
Nanos1B
Nanos1B Osteichthyes
Nanos1B Scyliorhinus canicula
Nanos1B Rhincodon typus
Nanos2, Nanos3
Nanos Drosophila melanogaster
81
86
62
77
94
75
39
90
49
89
69
81
0.050
B
Acanthopterygians
Nanos1Ba Takifugu rubripes
Nanos1Ba Epinephelus coioides
Nanos1Ba Oreochromis niloticus
Nanos1Ba Oryzias latipes
Nanos1Bb Oryzias latipes
Nanos1Bb Oreochromis niloticus
Nanos1Bb Takifugu rubripes
Nanos1Bb Epinephelus coioides
Cypriniformes
Nanos1Ba Sinocyclocheilus anshuiensis
Nanos1Ba Sinocyclocheilus rhinocerous
Nanos1Bb Danio rerio
Nanos1Bb Sinocyclocheilus anshuiensis
Nanos1Bb Sinocyclocheilus rhinocerous
Salmoniformes
Nanos1Ba Oncorhynchus mykiss
Nanos1Ba Salmo salar
Nanos1Bb Oncorhynchus mykiss
Nanos1Bb Salmo salar
Osteoglossiformes
Nanos1Ba Scleropages formosus
Nanos1Bb Scleropages formosus
Nanos1B Lepisosteus oculatus
51
69
100
54
79
83
73
92
97
69
89
53
96
100
83
23
81
0.050

Supplementary Figure S3: Phylogenetic trees illustrating the evolutionary relationship between the Nanos sequences.
Nanos protein sequences were aligned using BioEdit ClustalW multiple alignment editor and phylogenetic trees were built using the Molecular Evolutionary Genetics Analysis (MEGA) software version 7.0. Trees were constructed using the Neighbour-Joining method and the reliability of the inferred trees was assessed using the bootstrap procedure with 1000 replications. The scale bar indicates the number of expected amino acid substitutions per site per unit of branch length. For each species and each Nanos paralogous protein, sequence accession numbers were listed in Supplementary Table S5. The phylogenetic tree was rooted using the drosophila Nanos sequence (A). Sequences corresponding to Nanos2 and Nanos3 in different species have been compressed to focus the study on Nanos1 sequences. Chondrichthyan and osteichthyan Nanos1 sequences segregate together but two distinct Nanos1A and Nanos1B clusters (shaded boxes) are observed. Due to their high inter-species conservation, Nanos1B sequences of Osteichthyes were compressed in this global tree. Another secondary phylogenetic tree was built to investigate further the *nanos1B* gene duplication in Teleosts (B). This tree was rooted using the Nanos1B protein sequence of a Lepisosteiforme, the spotted gar, an out-group species to Teleosts which did not undergo the Teleost specific third whole genome duplication (termed 3R). Nanos1Ba and Nanos1Bb sequences segregate together inside different clades: Acanthopterygians, Cypriniformes, Salmoniformes and Osteoglossiformes respectively (shaded boxes) suggesting that *nanos1Ba* and *nanos1Ba* are ohnologs resulting from the *nanos1B* duplication. Note that no zebrafish *nanos1Bb* gene was identified from transcript (mRNA, EST, RNAseq) or genome libraries.

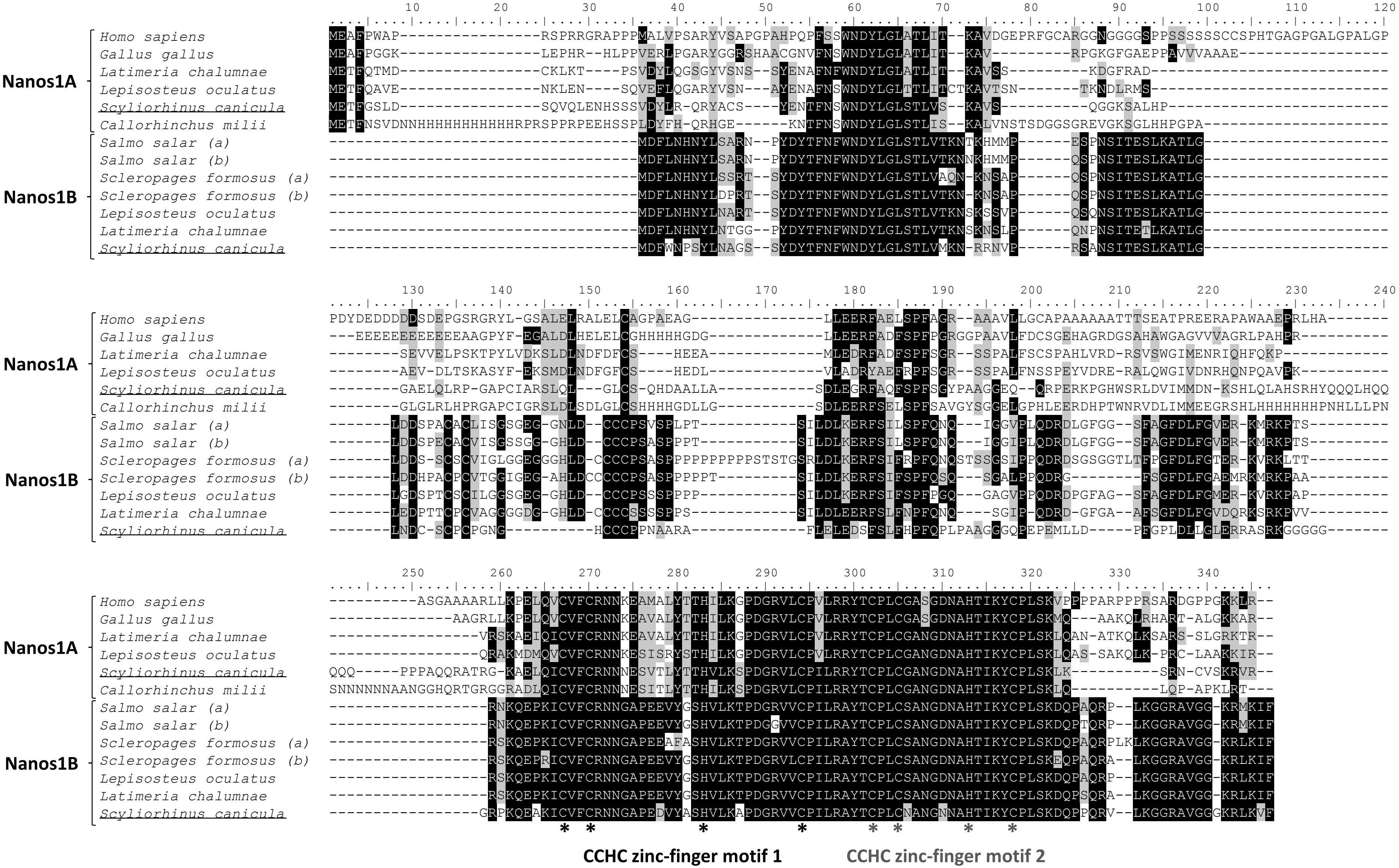
Nanos1A
Homo sapiens
Gallus gallus
Latimeria chalumnae
Lepisosteus oculatus
Scyliorhinus canicula
Callorhinchus milii
Nanos1B
Salmo salar (a)
Salmo salar (b)
Scleropages formosus (a)
Scleropages formosus (b)
Lepisosteus oculatus
Latimeria chalumnae
Scyliorhinus canicula
CCHC zinc-finger motif 1
CCHC zinc-finger motif 2

Supplementary Figure S4: Amino acid alignment of Nanos1 sequences reveals a higher conservation of Nanos1B sequences compared to Nanos1A.

Nanos1 complete protein sequences of representative vertebrate species were aligned using BioEdit ClustalW multiple alignment editor. Identical and similar residues are shaded in black and grey respectively following a 40% threshold. Nanos1A and Nanos1B are both detectable in chondrichthyan species such as dogfish and osteichthyan species such as coelacanth and spotted gar whereas the second duplicated gene copy (Nanos1Ba/b) is restricted to teleostean fish such as arowana and salmon, which have lost Nanos1A. The accession numbers of the selected proteins are detailed in Supplementary Table S5. The two Nanos specific CCHC zinc finger motifs are indicated at the carboxy terminal end of Nanos1A and Nanos1B proteins (black asterisks, zinc finger motif 1; grey asterisks, zinc finger motif 2). Nanos1B proteins show a higher conservation compared to Nanos1A proteins. For example, coelacanth Nanos1A presents only a mean of 7% identity and 16% similarity with the other selected Nanos1A whereas Nanos1B presents respectively 34% identity and 43% similarity with the other selected Nanos1B sequences.

Supplementary Table S5: Accession numbers of the Nanos protein sequences used for the realization of the phylogenetic trees.

The Nanos proteins sequences were predicted from the NCBI genome databases, except the coelacanth (*Latimeria chalumnae*) Nanos3 protein which was uploaded from the Ensembl genome browser. Concerning the dogfish (*Scyliorhinus canicula*), genomic scaffolds were deposited on NCBI and annotated.

species	Nanos(1A)	Nanos1B(a)	Nanos1Bb	Nanos2	Nanos3
Anguilla anguilla		scaffold_2622	scaffold_332	scaffold_4180	
Callorhinchus milii	XP_007901835.1			XP_007884862.1	NW_006890233*
Chelonia mydas	XP_007064298.1			XP_007071042.1	XP_007066396.1
Danio rerio			NM_001305661.1	DAA64468.1	NP_571953.1
Drosophila melanogaster	NP_476658.1				
Epinephelus coioides		ARU80562.1	ARU80563.1	ARQ20695.1	ARU80564.1
Gallus gallus	XP_015144398.1				
Homo sapiens	NP_955631.1			NP_001025032.1	NP_001092092.1
Latimeria chalumnae	XP_005999283.1	XP_006011198.1		XP_005996108.1	ENSLACP00000015098
Lepisosteus oculatus	XM_006630597.2	XP_015199289.1			
Oncorhynchus mykiss		XP_021439740.1	XP_021417026.1	XP_021428629.1	XP_021423321.1
Oreochromis niloticus		XP_005467279.1	XP_003447814.1	XP_005448912.2	XP_005460610.1
Oryzias latipes		AB437935.1	NM_001160469.1		
Rhincodon typus	XP_020378660.1	XP_020378957.1		XP_020380207.1	NW_018035638.1
Salmo salar		XP_014067484.1	NM_001141585.1	XP_014033933.1	AGG35558.1
Scleropages formosus		XP_018581993.1	XP_018601890.1		XP_018620400.1
Sinocyclocheilus anshuiensis		XP_016329477.1	XP_016305716.1	XP_016311246.1	XP_016361995.1
Sinocyclocheilus rhinocerous		XP_016377827.1	XP_016370734.1	XP_016397459.1	XP_016417115.1
Scyliorhinus canicula	MH015179	MH015180		MH015181	MH015182
Takifugu rubripes		XP_011618819.1	XP_011609291.1	XP_011606249.1	

* Not annotated

Supplementary Table S6: Nucleotide sequences of the primer sets used for real-time quantitative PCR and riboprobes synthesis. Primer sequences and PCR product sizes are indicated for each gene. A cDNA library previously established [21] was used with M13Fwd/Rev primers to produce riboprobes for *in situ* hybridization.

experimental procedure	gene name	GenBank accession number	primer name	primer sequence (5'-3')	PCR product size (bp)
RT-PCR	*5S rRNA*	M24954.1	Sc_5S rRNA_1q<br>Sc_5SrRNA_1Aq	TCGTCTGATCTCGGAAGCTA<br>AGCCTACTGCACCTGGTATTC	85
	nanos1A	MH015175	Nanos1Qfw1<br>Nanos1aQrv1	ATGGAAACTTTCGGGTCACT<br>GCTCTTCAGTTTGGAGAGTGG	131
	nanos1B	MH015176	Nanos1bQfw2<br>Nanos1bQrv2	CCGGGAGCAGTTACGATTACA<br>ACCTTCAGTCTCTTGCCCC	80
ISH	*nanos1A*	MH015177	M13 Fwd31	CAGTCACGACGTTGTAAAACGACGGCCAGTG	792
	nanos1B	MH015178	M13 Rev33	CAGGAAACAGCTATGACCATGATTACGCCAAGC	2815
